# Helix Folding in One Dimension: Effects of Proline Co-Solvent on Free Energy Landscape of Hydrogen Bond Dynamics in Alanine Peptides

**DOI:** 10.3390/life15050809

**Published:** 2025-05-19

**Authors:** Krzysztof Kuczera

**Affiliations:** 1 Department of Chemistry, The University of Kansas, Lawrence, KS 66045, USA; kkuczera@ku.edu; 2 Department of Molecular Biosciences, The University of Kansas, Lawrence, KS 66045, USA

**Keywords:** alanine peptides, hydrogen bond dynamics, proline co-solvent, helix folding

## Abstract

The effects of proline co-solvent on helix folding are explored through the single discrete coordinate of the number of helical hydrogen bonds. The analysis is based on multi-microsecond length molecular dynamics simulations of alanine-based helix-forming peptides, (ALA)n, of length n = 4, 8, 15 and 21 residues, in an aqueous solution with 2 M concentration of proline. The effects of addition of proline on the free energy landscape for helix folding were analyzed using the graph-based Dijkstra algorithm, Optimal Dimensionality Reduction kinetic coarse graining, committor functions, as well as through the diffusion of the helix boundary. Viewed at a sufficiently long time-scale, helix folding in the coarse-grained hydrogen bond space follows a consecutive mechanism, with well-defined initiation and propagation phases, and an interesting set of intermediates. Proline addition slows down the folding relaxation of all four peptides, increases helix content and induces subtle mechanistic changes compared to pure water solvation. A general trend is for transition state shift towards earlier stages of folding in proline relative to water. For ALA5 and ALA8 direct folding is dominant. In ALA8 and ALA15 multiple pathways appear possible. For ALA21 a simple mechanism emerges, with a single path from helix to coil through a set of intermediates. Overall, this work provides new insights into effects of proline co-solvent on helix folding, complementary to more standard approaches based on three-dimensional molecular structures.

## 1. Introduction

The focus of this work is modeling the folding of peptide helices in the presence of proline, using the number of hydrogen bonds as the reaction coordinate. Helices and sheets are crucial elements of protein structure, and they are characterized by characteristic hydrogen bond patterns [1]. Collective variables and reaction coordinates play a central role in models of protein folding, as they can be used to accelerate conformational sampling, calculate reaction rates and provide insight into microscopic mechanisms [2,3,4]. The fraction of native contacts and a number of geometrical coordinates have been employed in such studies [2,5]. Among the proposed geometric functions have been RMSD and average hydrogen length [5]. Here, we focus on the number of formed helical hydrogen bonds as a coordinate describing the folding of alpha helical peptides, and follow the effects of addition of the protective osmolyte proline at 2 M concentration.

Effects of osmolytes in general and proline in particular have been the topic of a wide range of experimental and computational studies [6,7,8,9]. In experimental studies and the transfer model of osmolyte action, proline has been determined to stabilize folded forms of proteins and helices [10,11,12,13,14]. It tends to be excluded from the solute solvation shell, and induce its compaction and de-solvation [15]. The choice of 2 M proline in our study is based on experimental and computational considerations. From the computational standpoint, the concentration must be high enough to observe a statistically significant effect of osmolyte addition, but low enough for efficient sampling of conformational transitions. Experimentally, a range of concentrations has been explored, with strongest effects reported at 2 M proline [11].

Previously, we have performed extensive simulations for alanine-based peptides of length 5, 8, 15 and 21 [16]. From long-term MD simulations in aqueous solution, we predicted helix fractions, folding times and microscopic folding paths that were in reasonable agreement with available experimental data and previous simulations. Surprisingly, a simplified coarse-grained treatment based on following the number of formed helical hydrogen bonds was able to reproduce the main elements of the multidimensional structure-based analysis [17]. More recently, we have studied the effects of addition of the osmolyte proline as co-solvent on the structure, dynamics and interactions on the same alanine peptides as studied here [18]. Based on analysis of 3D structures we found a stabilization of the helical state and slowing down of the kinetics, as well as changes in the folding pathways [18]. Additionally, microscopic effects of exclusion of the proline from the solute region, as well as peptide de-solvation and compaction were observed [18]. Thus, addition of proline to the solution had profound macroscopic and microscopic effects on folding [18,19].

This work presents an analysis of peptide helix folding based on hydrogen bond breaking and formation in the presence of 2 M proline co-solvent. For four alanine-based peptides, ALA5, ALA8, ALA15 and ALA21, we employ previously generated microsecond length MD trajectories in 2 M proline solution [18] to analyze hydrogen bonding patterns. Folding paths in hydrogen bond space are analyzed through coarse-grained kinetic models, graph-based global minimum weight paths, Transition Path Theory and helix boundary diffusion.

The stabilization of the helix and slowing down of folding/unfolding relaxations due to addition of proline have a marked influence on the free energy landscape of the studied peptides. This is reflected in changes in microstate populations, kinetic rate constants, transition paths and helix boundary diffusion. Interestingly, proline effects vary markedly with peptide length. Except for ALA5, there are definite shifts of population from states with lower to those with higher numbers of helical hydrogen bonds (NHB). The microscopic reaction rate constants for hydrogen bond transitions are systematically lower in 2 M proline relative to water. Graph-based analysis of maximum flux paths shows shifts in the path bottlenecks relative to water. Transition states for folding tend to correspond to earlier stages of helix formation in proline. In coarse-grained kinetic models based on Optimal Dimensionality Reduction (ODR) [20], the direct folding mechanism is found for ALA5, ALA8 and ALA15 with proline, but only for the first two in water. For ALA21, the presence of proline greatly simplifies the folding mechanism, which becomes a simple linear folding along a single path, with systematic increase in hydrogen bond count. Helix boundary diffusion is also found to be slower in 2 M proline relative to water. The strongest influence of proline presence is seen in the case of ALA15—this peptide has largest increases of helix content and slowing of folding relaxation and helix boundary diffusion. Overall, the lens of hydrogen bond space paints a subtly different and complementary picture of proline effects on helix folding compared to the more standard results of structure-based kinetic modeling [18].

## 2. Methods

Four blocked peptides, Ac-Ala_5_-NH_2_ (ALA5), Ac-Ala_8_-NH_2_ (ALA8), Ac-Ala_15_-NH_2_ (ALA15), and Ac-Ala_21_-NH_2_ (ALA21), were studied. Molecular dynamics (MD) simulations were performed at 300 K in explicit water and Na^+^ and Cl^−^ ions at total 0.15 M concentration and 2.0 M proline, as described previously [18]. For the shorter peptides, ALA5 and ALA8, two independent trajectories, starting from the helical (trajectory a) and extended (trajectory e) structures were generated. The trajectories were 5 μs each for ALA5 and 10 μs each for ALA8. For the longer peptides, five independent trajectories were generated, denoted a–e, of 10 μs each for ALA15, and 20 μs each for ALA21 [18]. The MD simulations were performed with GROMACS 5.1.4 [21] using the CHARMM36m [22] force field and TIP3P [23] water model. A time step of 2 fs was employed, with constant number of particles, volume and temperature (NVT) conditions, with the temperature of 300 K maintained by velocity scaling. Nonbonded cutoffs were 1.2 nm, and the Particle-Mesh Ewald (PME) [24] method was used to account for long-range electrostatic interactions. Trajectory details may be found in ref. [18] and in the SI (p. 1 and p. 24).

**Figure 1:**
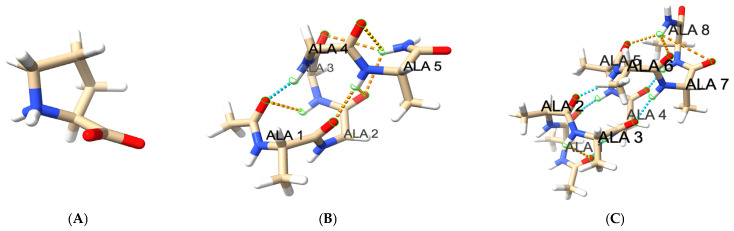

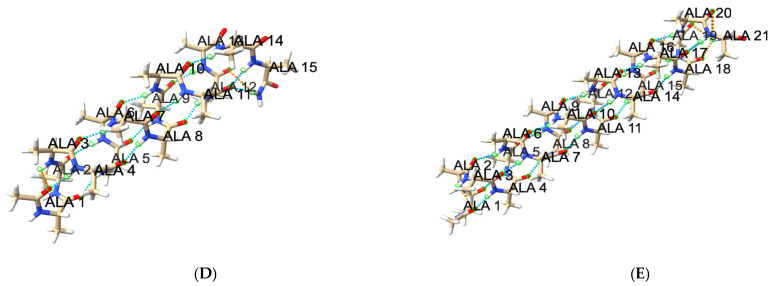
Molecular structures of simulated systems. (**A**) Proline zwitterion with NH_2_^+^ N-terminus and COO^−^ C-terminus, neutral overall. (**B**) ALA5 helix (**C**) ALA8 helix. (**D**) ALA15 helix. (**E**) ALA21 helix. Alanine peptides include N-terminal acetyl blocking group and C-terminal amide blocking group and are neutral overall. The hydrogen bonds considered here involve CO of residue i and HN of residue i + 4, starting with acetyl C=O to HN of Ala 4, and end with Ala (n − 3) C=O to H_2_N of amide blocking group, with n = 5, 8, 15, 21. Standard hydrogen bonds are shown in blue; some distorted hydrogen bonds present at termini shown in orange.

Helical hydrogen bonds length time series were calculated for all peptide C=O oxygens of residue i and the peptide N-H nitrogens of residue i + 4. In order to smooth out the data, each hydrogen bond was counted as fully formed for O…N distance below 3.2 Å (weight of 1) and fully broken for distances above 4.0 Å (weight of 0). At intermediate distances the bond was partially formed, with an intermediate weight obtained from a cubic interpolation. The weights for all helical hydrogen bonds were then added up in order to obtain the value of the reaction coordinate, NHB—the total number of present alpha helical hydrogen bonds. Our blocked peptides had maximum numbers of helical hydrogen bonds (MAXHB) of 3, 6, 13, and 19 for ALA5, ALA8, ALA15, and ALA21, respectively. The helix fraction f for each trajectory was calculated as the trajectory average of the ratio NHB(t)/MAXHB, where NHB(t) is the number of formed helical h-bonds at time t. The structures of the helical conformations of the peptides are shown in Figure 1, which also illustrates the hydrogen bonds present and the structure of proline.

As described in the SI (pp. 1–2) and previously, the dynamical time scales are calculated from fits of autocorrelation functions to two-exponential decays, which yield a shorter time scale $\tau_{1}$ and a longer time scale $\tau_{2}$. The short time scale is assigned to local fluctuations and the long one to global folding. The folding equilibrium constant $K=f/\left(1-f\right)$, obtained from the helix fraction f, is used to calculate the folding and unfolding times from the $\tau_{2}$ relaxation time as

(1) $$k_{u}=\frac{1}{\tau_{2}}\frac{1}{1+K}\;and\;k_{f}=\frac{1}{\tau_{2}}\frac{K}{1+K}$$

Kinetic modeling was performed in the space of helical hydrogen bonds, defined by the MAXHB + 1 microstates with NHB = 0, 1, 2, …, MAXHB, and has dimension 4 for ALA5 (NHB from 0 to 3), 7 for ALA8 (NHB from 0 to 6), 14 for ALA15 (NHB from 0 to 13) and 20 for ALA21 (NHB from 0 to 19). The trajectories were discretized by assigning each time frame to the closest microstate. Initial kinetic matrices and transition matrices were calculated from transitions and residence times in the NHB space, using the moving window count method, based on data processing used in Markov State Modeling [25]. The lag time was chosen so that the slowest relaxation agreed with the slow correlation time of global properties from MD (see SI Tables S2–S5 for details). Finally, kinetic coarse-graining was performed using PCCA+ [26], to lower dimensionality further to 2–4 dimensions, and effective rates determined with optimal dimensionality reduction (ODR) [20,27]. Committor values q were calculated with the EMMA v.1 [28] package transition path theory tool.

Global maximum weight paths (GMWPs) in hydrogen bond space were calculated using the recursive Dijkstra algorithm of Elber et al. [29], with initial state NHB = 0 and final state NHB = MAXHB for each peptide. The total number of transitions between microstates, proportional to the reactive flux, symmetrized to ensure detailed balance were used as edge weights (see SI Tables S6a–d and S7a–d).

Diffusion and friction for helix folding were obtained using the approach of Bicout and Szabo [30], based on microstate rate constants and populations, as described previously. Details are in Figure S1.

Molecular graphics in Figure 1 were generated with Chimera-X v.1.7.1 [31].

## 3. Results and Discussion

The detailed 3D-structure-based analysis of structure, folding and interactions of the ALA5-ALA21 peptides has been described in detail previously both in aqueous solution [16] and in the presence of 2 M proline co-solvent [18]. Briefly, the helix fractions measured by hydrogen bond counts were 3%, 6%, 25% and 53% in water [16] and 3%, 11%, 49% and 68% in 2 M proline [18], for ALA5, ALA8, ALA15 and ALA21, respectively. The corresponding simulated relaxation times for the folding equilibrium were 2.4, 13, 110 and 340 ns in water [16] and 3, 34, 540 and 460 ns in 2 M proline [18]. The main conclusions are that both helix content and relaxation times increase with peptide length, and that the addition of proline stabilizes the helical state and slows down the folding/unfolding processes. In this work, the same MD trajectories that were the basis of folding analysis in multidimensional conformational space [18] are used to view the same process through the one-dimensional picture of total number of helical hydrogen bonds, NHB. The molecular structures of proline and the alanine helices are shown in Figure 1.

The time scales of peptide dynamics determined with different approaches are presented in Table 1. The MD data exhibit a wide range of time scales. The slowest MD time scale $\tau_{2}$ can be well determined based on analysis of several structural quantities. By setting values of model parameters, the cluster core radius for RMSD clustering and lag time for NHB transitions, the slow times from RMSD and NHB kinetic models have been adjusted to agree with MD results. The kinetic models are designed to represent the slowest dynamical processes in our systems.

Due to presence of many fast processes, the determination of the fast time scale $\tau_{1}$ directly from MD is approximate. The MD $\tau_{1}$ timescales are assigned to individual hydrogen bond fluctuations, and are more than an order of magnitude faster than the MD $\tau_{2}$ values. Due to the coarse-graining used in the kinetic models, the fast time scales in RMSD and NHB analysis are much longer than in MD and typically represent transitions involving forming/decay of folding intermediates (see below). In the case of the low dimensional NHB models, the values are equal to the lag times used in transition calculations, so they represent the lower end of the model time resolution.

### 3.1. Microstate Populations

**Figure 2:**
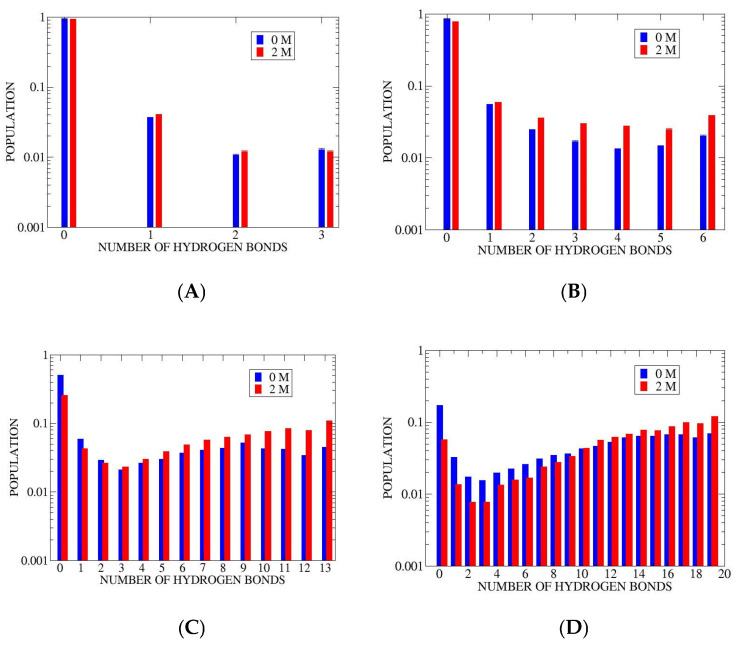
Hydrogen bonding microstate population comparison between aqueous solution (0 M) [16] and 2 M proline solution (2 M). Results averaged over independent trajectories, two for ALA5 and ALA8, and five for ALA15 and ALA21. (**A**) ALA5 (**B**) ALA8 (**C**) ALA15 (**D**) ALA21. Statistical errors are about 5–10% of values. Logarithmic scale is used.

Populations of the individual hydrogen bonding count states are given in Figure 2. The data for 2 M proline solutions are compared to results from aqueous simulations [16]. For ALA5 the effect of proline is small. In the longer peptides, ALA8-ALA21, there is a systematic trend of population shift from states of lower to higher NHB counts. This shift is reflected in overall increases in helix population in 2 M proline.

### 3.2. Folding Kinetics in Hydrogen Bond Space

Kinetic rates $k_{ij}$ describing j→*i* transitions between microscopic states are presented in Table 2 for ALA5, Table 3 for ALA8, with remaining data in SI (Tables S2–S5).

The ALA5 kinetic matrix in Table 2 shows much higher rates of unfolding (upper triangle) relative to folding (lower triangle). Since rate constants $k_{ij}$ and populations $p_{i}$ are related by the detailed balance condition $k_{ij}p_{j}$ = $\;k_{ji}p_{i}$, this may be related to the lower populations of the states with higher NHB. Forward and reverse transitions occur at similar rates between states 2 and 3, which have comparable populations (Figure 2A). Also, there is a trend for faster transitions in processes involving smaller changes of the NHB variable, with rate constants decreasing away from the main diagonal. The fastest processes involve formation of the coil (NHB = 0)—breaking of the last hydrogen bond, 1→0 with rate constant 375 ${\mu s}^{-1}$, followed by 2→0 with 246 ${\mu s}^{-1}$ and 3→0 at 207 ${\mu s}^{-1}$. In the case of ALA5 transitions between all microstates are found, including direct folding 0→3 (at 2.6 ${\mu s}^{-1}$) and unfolding 3→0 (at 207 ${\mu s}^{-1}$).

The full kinetic matrix for ALA8 transitions in hydrogen bond space is shown in Table 3. Here also the trend for faster transitions with smaller changes in NHB is evident. The fastest processes involve the final stages of helix unfolding, with rate constants of 93 ${\mu s}^{-1}$ for 1→0 and 66 ${\mu s}^{-1}$ for 2→0. Early stages of helix unfolding tend to exhibit similar forward and reverse rate constants, e.g., 30 ${\mu s}^{-1}$ for 6→5 and 44 ${\mu s}^{-1}$ for 5→6. For ALA8 all possible microstate transitions are observed. This includes direct folding 0→6 at 0.3 ${\mu s}^{-1}$ and unfolding 6→0 at 7.1 ${\mu s}^{-1}$, although these processes are relatively slow.

The kinetic matrices for ALA15 and ALA21 are shown in Tables S4 and S5. For these longer peptides, the fastest transitions also involve the final stages of helix unfolding. The rate constants for 1→0, 2→0 and 3→0 transitions are 5.6, 5.1 and 4.4 ${\mu s}^{-1}$ for ALA15 and 6.6, 5.2 and 3.6 ${\mu s}^{-1}$ for ALA21. Here also, the transitions among states with highest numbers of hydrogen bonds are relatively symmetrical, e.g., rate constants for 14→13 and 13→14 are 1.5 and 1.7 ${\mu s}^{-1}$ in ALA15, and constants for 19→18 and 18→19 are 2.3 and 2.1 ${\mu s}^{-1}$ in ALA21. The slower rates of transitions for ALA15 relative to ALA21 are in accord with the calculated relaxation times, 540 ns for ALA15 vs. 470 ns for ALA21 [18]. For ALA15 and ALA21 we do not detect direct helix-coil transitions in the full space of all microstates.

The main features in the $k_{ij}$ data are that rate constants are lower for the longer peptides and that the fastest processes involve the final stages of coil formation. The rates are fastest for transitions involving breaking/formation of single hydrogen bonds. Finally, direct helix-coil transitions are found for ALA5 and ALA8 only.

### 3.3. Global Maximum Weight Paths (GMWP)

GMWPs in the space of hydrogen bond count microstates are calculated using the recursive Dijkstra algorithm described in [29], using symmetrized total transition counts as weights [18]. Briefly, for any path between initial state *i* and final state *f*, the bottleneck is defined as the edge with minimum weight. A maximum weight path (MWP) between *i* and *f* has the highest bottleneck weight, while in a GMWP all sub-paths are also MWPs.

**Figure 3:**
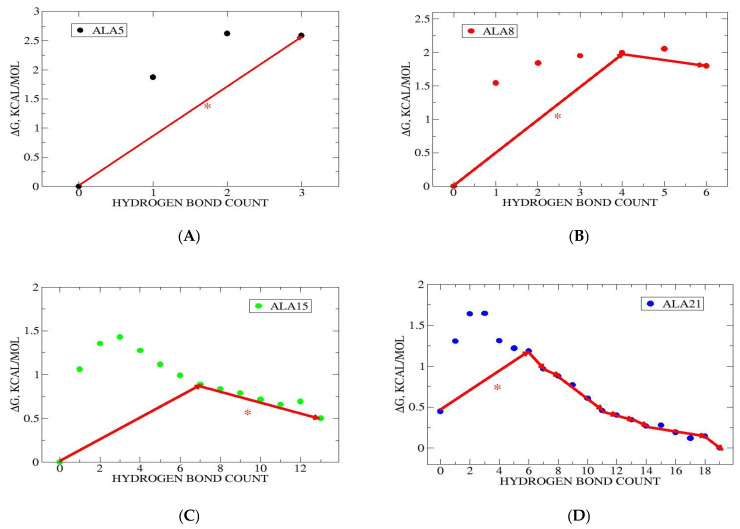
Global maximum weight paths in the space of helical hydrogen bond counts. State populations shown as circles with free energies, $∆G_{i}=-RT\ln\frac{p_{i}}{p_{max}}$, with R the gas constant, T = 298 K, $p_{i}$ the population of state *i* and $p_{max}$ the maximum population for a given peptide. GMWPs are shown by arrows and the bottlenecks marked by asterisk, “*”. (**A**) ALA5 GMWP is 0→3, same as bottleneck. (**B**) ALA8 GMWP is 0→4→6, with 0→4 bottleneck. (**C**) ALA15 GMWP is 0→7→13, with bottleneck 7→13. (**D**) ALA21 GMWP is 0→6→7→8→11→12→13→14→18→19, with bottleneck 0→6.

Thus, the GMWP is a path through the microstates that will have the highest reactive flux. The GMWPs for helix folding of the four studied peptides are shown in Figure 3.

For ALA5, the GMWP is 0→3, which is also the bottleneck. This indicates the preference for direct coil→helix folding, without intermediates. In the case of ALA8, the GMWP is 0→4→6, indicating the presence of an intermediate with NHB = 4 hydrogen bonds formed. The bottleneck edge is 0→4, i.e., formation of NHB = 4 intermediate from coil. In the case of ALA15, the GMWP is 0→7→13, showing the presence of an intermediate with NHB = 7. Here the bottleneck is 7→13, i.e., formation of the helix at NHB = 13 from the NHB = 7 intermediate. For ALA21, the GMWP follows states 0→6→7→8→11→12→13→14→18→19, with eight intermediates. The bottleneck 0→6, describing formation of the first intermediate, with NHB = 6, from the coil, NHB = 0.

Generally, the number of intermediates increases with peptide length. ALA5 exhibits direct folding, ALA8 and ALA15 pass through a single intermediate, while ALA21 has multiple intermediates on its folding path. The bottlenecks involve early stages of folding for ALA8 and ALA21, and late stages for ALA15.

The pathways describe consecutive folding in 2 M proline, with monotonic increases in the NHB variable, which was also the case for hydrogen bond dynamics in water [17]. In the cases of ALA5 and ALA8, the free energy landscapes and GMWPs are similar in 2 M proline and water [17]. For ALA15 and ALA21, there are significant shifts in microstate populations toward higher helicity in 2 M proline as compared to water. This is accompanied by changes in folding paths. The biggest change is for ALA15, where the GMWP in 2 M proline is significantly simplified compared to water, with one intermediate in the proline and eight in the aqueous simulations [17]. Further, the bottleneck is shifted from 0→5 in water to 7→13 in proline. For ALA21, the number of intermediates increases from seven in water to eight in proline, with some differences in the NHB values of the intermediate states. Also, the bottleneck shifts from 0→9 in water to 0→6 in proline, moving toward earlier helix formation stages.

#### 3.3.1. Coarse-Grained Kinetic Models

Based on the discretized trajectories in hydrogen bond space, we performed kinetic coarse-graining using Optimal Dimensionality Reduction (ODR) [20,27], obtaining kinetic models of dimension N = 2–4 for the peptides. Details are described in the SI (Tables S6a–d and S7a–d). The ODR approach is designed to provide a simplified description of the slowest dynamical processes in the system, easing structural interpretation of motions. In order to eliminate fast fluctuations, transitions in the microstate space are counted with a lag time $\tau_{l}$ [17,25]. For each peptide, the appropriate $\tau_{l}$ is chosen so that the slowest ODR relaxation time matches the slowest MD dynamical time scale $\tau_{2}$ (see Table 1 above). The lag times were 2, 8, 100 and 80 ns for ALA5, ALA8, ALA15 and ALA21, respectively. The slowest ODR relaxation times were within 30% of the MD results (Table 4 and Table S6a–d). In all the models, the aggregate state containing the NHB = 0 microstate is assigned as the coil, the state containing NHB = MAXHB microstate is assigned as the helix, and the rest are classified as intermediates.

**Figure 4:**
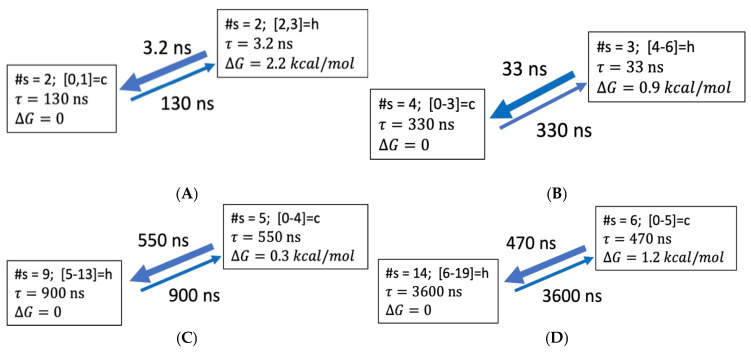
Coarse-grained kinetic models of dimensionality N = 2 based on hydrogen bond microstate transitions in 2 M proline. Notation for coarse-grained state descriptions: #s—number of microstates belonging to aggregate state; e.g., [0, 1]—state consisting of NHB = 0 and NHB = 1 microstates; h—helix; c—coil. (**A**) ALA5 model based on discretization with lag time $\tau_{l}=2.0\;\mathrm{ns}$. (**B**) ALA8 model with $\tau_{l}=8.0\;\mathrm{ns}$. (**C**) ALA15 model with $\tau_{l}=100\;\mathrm{ns}$. (**D**) ALA21 model with $\tau_{l}=80\;\mathrm{ns}$.

Two state models. The ODR kinetic models of dimension N = 2 are summarized in Figure 4 and Table 4. Two main features of the models are consecutive folding paths and heterogenous nature of both the helix and coil aggregate states. The folding and unfolding times, as well as helix stability, all tend to increase with peptide length. Table 4 compares the two-state kinetic model parameters obtained directly from MD helix fractions and relaxation times (Equation (1)) and through ODR calculations of dimension N = 2. The folding and unfolding times, as well as the folding free energies from the two approaches are similar, though not identical. The largest deviations are for the ALA5 folding and ALA21 unfolding times. Also, the ALA15 helix stability and unfolding time appear to be overestimated by ODR. Thus, the dimension N = 2 ODR models do not capture the full complexity of the structural fluctuations, due to the coarse graining.

**Figure 5:**
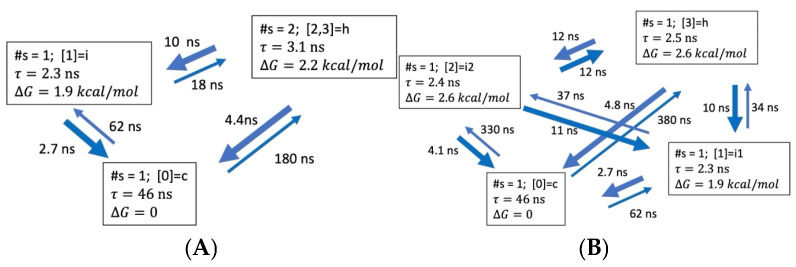
Coarse-grained kinetic models for ALA5 of dimensionality N = 3, 4 in 2 M proline, based on transitions between four microstates, NHB = 0, 1, 2, 3, 4 and lag time $\tau_{l}=2.0\;\mathrm{ns}$ (**A**) ODR dimension N = 3. (**B**) ODR dimension N = 4. More details are in SI Table S7a.

ALA5. The more detailed ODR models for ALA5 are shown in Figure 5. The helix population remains low in all models, with ∆G of 2.2–2.6 kcal/mol above the coil. For N = 3 and N = 4, intermediates become populated. As in the full kinetic matrix (Table 2), the fastest processes are forming the coil from the intermediates. Direct folding is also quite fast, with time scales of 4.4 ns in N = 3 and 4.8 ns in N = 4. Alternative folding paths are slower.

**Figure 6:**
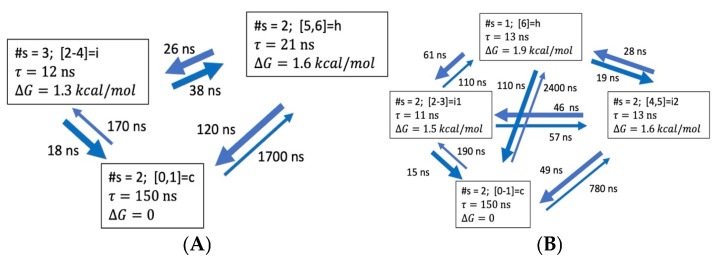
Coarse-grained kinetic models for ALA8 of dimensionality N = 3, 4 in 2 M proline, based on transitions between seven microstates, NHB = 0, 1, …, 6 and lag time $\tau_{l}=8.0\;\mathrm{ns}$. (**A**) ODR dimension N = 3. (**B**) ODR dimension N = 4. More details are in SI Table S7b.

ALA8. The N = 3, 4 ODR models for ALA8 are shown in Figure 6. In this case, the helix population is low as well, with ∆G of 1.6–1.9 kcal/mol above the coil. The helix state is represented by NHB = 5–6 in the N = 3 and NHB = 6 only in the N = 4 model, while the coil aggregate includes NHB = 0, 1 in both cases. Again, the fastest processes are transitions from intermediates to the coil, at 15–50 ns. The direct folding transition is present, with a time constant of 110–120 ns. Multiple folding paths become feasible.

**Figure 7:**
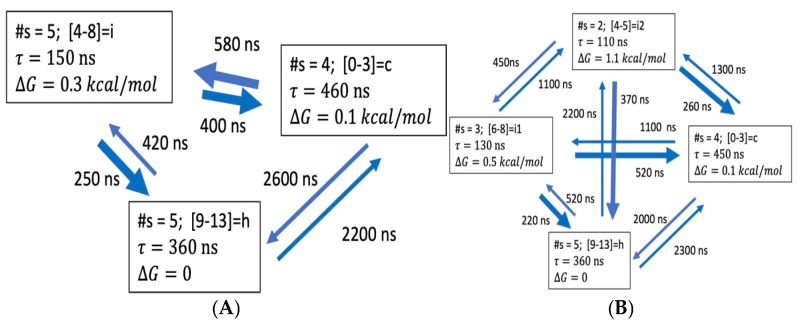
Coarse-grained kinetic models for ALA15 of dimensionality N = 3, 4 in 2 M proline, based on transitions between fourteen microstates, NHB = 0, 1, …, 13 and lag time $\tau_{l}=100\;\mathrm{ns}$. (**A**) ODR dimension N = 3. (**B**) ODR dimension N = 4. More details are in SI Table S7c.

ALA15. Figure 7 presents the N = 3, 4 ODR models for ALA15. All aggregate states are heterogenous, involving several microstates. These are NHB = 9–13 for helix and NHB = 0–3 for coil for both N = 3 and N = 4. At the microstate level, the coil is the most populated (Figure 2) and direct folding is not detected (SI, Table S4). In the coarse-grained picture, we find that the helix aggregate is most stable and a slow direct helix-coil transition is present, due to heterogeneity of the aggregates. The coil is only 0.1 kcal/mol over the helix state. The intermediates are 0.3–1.1 kcal/mol above the helix. Multiple folding paths are present.

**Figure 8:**
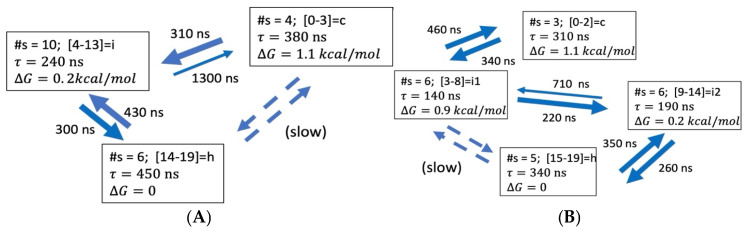
Coarse-grained kinetic models for ALA21 of dimensionality N = 3, 4 in 2 M proline, based on transitions between twenty microstates, NHB = 0, 1, …, 19 and lag time $\tau_{l}=80\;\mathrm{ns}$. (**A**) ODR dimension N = 3. (**B**) ODR dimension N = 4. More details are in SI Table S7d.

ALA21. The N = 3, 4 ODR models for ALA21 are shown in Figure 8. In this case, the helix state is the stable form. As a result, the folding times tend to be shorter than the unfolding times. The helix consists of microstates 14–19 in N = 3 and 15–19 in N = 4 models. For the coil, the microstates are 0–3 in N = 3 and 0–2 in N = 4. For ALA21, the models predict a single major folding path, with consecutive increases in NHB, passing through one (N = 3) or two (N = 4) intermediates.

In summary, the kinetic modeling indicates that the slowest processes are transitions involving systematic increases in the number of helical hydrogen bonds NHB. For the shorter peptides, ALA5 and ALA8, direct helix-coil folding is an effective process. In ALA5-ALA15, multiple pathways are possible. For ALA21, a single consecutive folding path emerges, with intermediates spanning microstates 4–13 in N = 3, and 3–8 and 9–14 in N = 3 models.

#### 3.3.2. Transition Path Theory

Analysis of the microstate and ODR kinetic networks allowed determination of committor functions q, using Transition Path Theory (TPT) [25,32,33,34], with details shown in SI Tables S8a–d (pp. 14–16). Especially interesting is the ability to detect transition states (TS), defined as states with q = 0.5. For ALA5, no transition states were identified in either 2 M proline or water [17]. In ALA8, states with q ≈ 0.5 corresponded to NHB = 5 in 2 M proline, compared to NHB = 4 in water. In ALA15, the NHB = 8 state was closest to the transition state, and it was NHB = 7 in water. In the case of ALA21, the TS predictions varied with model dimensionality. In the ODR models transition states corresponding to aggregates with intermediate hydrogen bond numbers were found, e.g., NHB = 3–8 in 2 M proline and NHB = 3–9 in water for N = 4. In the full microstate space, the TS were approximately located at NHB = 5 in proline and NHB = 10 in water.

#### 3.3.3. Comparing Helix Folding Paths from GMWP, ODR and TPT

Both ODR and GMWP analyses predict progressive folding mechanisms, with systematic increases in NHB from coil to helix. The GMWPs indicate that folding proceeds though a single, direct step for ALA5, two steps for ALA8 and ALA15, and multiple stages for ALA21. The bottlenecks involve the initial stages of helix formation in ALA8 (0→4) and ALA21 (0→6), and the final stages (7→13) in ALA15.

For ALA5, both ODR and GMWP approaches predict direct coil-helix folding. In the ALA8 case, the 0→4 GMWP bottleneck is similar to the 2–3, 2–4 and 4–5 aggregate state intermediates in ODR. For ALA15, there is a significant difference between GMWP and ODR. ODR intermediates do involve the mid-range of NHB, but multiple pathways are present and it appears that the initial stages of folding are slower than the final ones. The GMWP shows a bottleneck at the later stages of folding. It appears that the GMWP, which chooses a single path, does not represent ALA15 folding well. For ALA21, there is good agreement, with single path sequential folding from GMWP and ODR mechanism passing through intermediates in the mid-range of the NHB variable.

The TPT transition states detected in proline were systematically shifted to lower NHB values relative to water. This is consistent with the overall pattern of helix stabilization by proline addition. For ALA8 and ALA21, the patterns of variation with peptide size for TPT transition states and GMWP bottlenecks were similar.

#### 3.3.4. Hydrogen Bond Patterns and Folding Intermediates

As described previously, there are n!n−k!k! arrangements, or patterns, for a helix with k formed hydrogen bonds out of n possible. The details of these patterns are described in Table 5, Table 6 and Table S9a–d [17]. The patterns are encoded here as strings of 0/1 characters, with ‘1’ denoting a formed and ‘0’ a broken hydrogen bond. Thus, the pattern ‘110’ for ALA5 (with 3 possible bonds) denotes a structure with formed hydrogen bonds at the N-terminus and center, and broken hydrogen bond at the C-terminus.

Patterns sampled for ALA5 are presented in Table 5 and those for ALA15 in Table 6, while ALA8 and ALA21 results are in Table S9c,d. The main features of the patterns are the extra stability of terminal partial helices, like ‘110’ and ‘001’ for ALA5 or ‘0000001111111’ and ‘1111111000000’ for ALA15, relative to other locations in the sequence. This is also true in the folding intermediates detected in the folding path analysis above. The higher stability of terminal helices was also seen in water [17]. The main difference between water and proline patterns is the enhanced probability of states with higher NHB counts in the presence of the proline co-solvent.

#### 3.3.5. Diffusion and Friction

**Figure 9:**
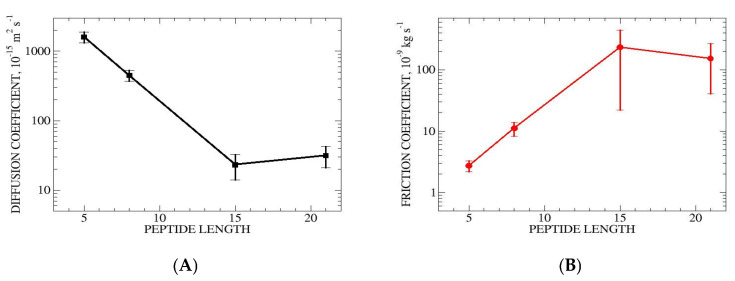
Average diffusion (**A**) and friction (**B**) coefficients for helix propagation in the four studied peptides. The values are averaged over the set of possible microstate transitions from i to i+1. Values for individual transitions are in Figure S1.

Diffusion D and friction f coefficients for helix propagation have been estimated using the method of Bicout and Szabo [30], as described in Section 2 and in the SI (Figure S1, p. 23). The position-dependent D and f values for transitions involving additions of individual hydrogen bonds are shown in Figure S1. These data show a minimum in D (and maximum in f) at early stages of folding, corresponding to addition of the first or second hydrogen bond. This reflects the high values of rate constants for these processes (Table 2, Table 3 and Tables S2–S5). The average D and f for the four peptides are shown in Figure 9. The average D values were 1600±300, 450±80, 23±9, and $32\;\pm 11\times 10^{-15}{\;m}^{2}s^{-1}$ for ALA5, ALA8, ALA15 and ALA21, respectively. The corresponding f values were 2.7±0.5, 11.0±2.7, 230±210 and $150\pm 110\times 10^{-9}\;{\mathrm{kg}\;s}^{1}$. Overall, there is a trend for slowing diffusion and increasing friction with increase in peptide length. The variation is approximately exponential. The D and f results for ALA15 are similar to those for the longer ALA21 peptide. This may be correlated with the significantly larger effect of proline on helix stability and folding relaxation time for the ALA15 system relative to the other peptides.

Comparing with aqueous solution simulations, the addition of 2 M proline has a small effect on D and f of ALA5 [17]. For ALA8 and ALA21, diffusion is slower (and friction higher) in 2 M proline, by about a factor of 2. The most dramatic effects are for ALA15, for which the D and f change by a factor of 20 relative to water.

#### 3.3.6. Effects of Proline on Helix Folding Free Energy Landscape

The effects of proline addition on molecular structure and overall relaxation have been described previously and summarized at the top of this section. Here we only present effects seen in the hydrogen bond microstate space. The stabilization of the helix and slowing down of folding/unfolding relaxations have a marked influence on the free energy landscape of the studied peptides. This is reflected in changes in microstate populations, kinetic rate constants, transition paths and helix boundary diffusion. Except for ALA5, there are definite shifts of population from states with lower NHB to those with higher NHB. The microscopic reaction rate constants for hydrogen bond transitions are systematically lower in 2 M proline relative to water. In the case of ALA5, the slowing down of folding and unfolding are the main effect, while more changes are found for the longer peptides. The Global Maximum Weight Paths in proline are subtly different, with bottlenecks shifted toward earlier stages of folding in ALA8 and ALA21, and toward later stages in ALA15. In the coarse-grained ODR kinetic models, the direct folding mechanism is found for ALA5, ALA8 and ALA15 in proline, but only for the first two in water. For ALA21, the presence of proline greatly simplifies the folding mechanism, which becomes a simple linear folding with systematic increase in hydrogen bond count, while multiple pathways appear in water. TPT analysis indicates that the transition states for folding are systematically shifted towards earlier stages of the process for ALA8-ALA21 in 2 M proline. Helix boundary diffusion is also found to be slower in 2 M proline relative to water. The strongest influence of proline presence is seen in the case of ALA15—this peptide has largest increases of helix content and slowing of folding relaxation and helix boundary diffusion.

#### 3.3.7. Comparison with Experimental Data

Helix content and folding times obtained for our peptides in aqueous simulations were in accord with available experimental data [16]. Experimentally, proline and other osmolytes are known to stabilize folded states of proteins and increase cooperativity of folding. Proline is specifically predicted to stabilize the folded state, slow down diffusion and form small self-aggregates [6,7,11]. The main effects found in the structure-based analysis of 2 M proline simulations of ALAn peptides [18]—increased helix content and slower relaxation—qualitatively agree with the empirical observations. Other microscopic effects found in simulations in the presence of proline [18] include osmolyte exclusion from peptide solvation shell, peptide compression and strong interactions with aromatic sidechains [9,15,35].

In this work, the focus is on helical hydrogen bonds, which involve the peptide backbone only, so the microscopic details of solvation are not directly addressed. However, many details of the folding process found here are similar to those emerging from the more detailed structural analysis performed previously. The hydrogen bond kinetic picture is similar to results of 3D structural analysis [18], and both are generally compatible with available experimental data. Thus, it appears that the helix folding landscapes presented here are based on structural and dynamical data that reliably represent the behavior of small peptides in the presence of 2 M proline. This is another example of realistic modeling of protein and peptide systems with CHARMM36m [22] potentials combined with the TIP3P [23] water model.

## 4. Conclusions

Based on multi-microsecond MD trajectories, effects of the presence of 2 M proline on helix folding were analyzed for four alanine peptides—ALA5, ALA8, ALA15 and ALA21. In this work, the focus was on dynamics along a single coordinate, the number of formed alpha-helical hydrogen bonds NHB. This discrete coordinate is described by a small number of microstates, four for ALA5, seven for ALA8, fourteen for ALA15 and twenty for ALA21. Peptide structures and dynamics were analyzed through calculation of microstate populations and transition rate constants, graph analysis, committors, kinetic coarse graining and modeling of helix boundary diffusion. Surprisingly, viewed at a sufficiently long time scale, helix folding in hydrogen bond space follows a consecutive mechanism, with well-defined initiation and propagation phases, and an interesting set of intermediates.

As described previously using conventional structural analysis, the presence of proline stabilizes the helical state and slows down the folding/unfolding relaxation [18]. Additional microscopic effects of proline included its exclusion from peptide solvation shell and increase in solution viscosity [18]. Peptide compaction and dehydration were also found, with stronger influence in the helix than in the coil [18]. All these changes have a marked influence on the hydrogen bonding free energy landscape of the peptides, reflected in changes in microstate populations, kinetic rate constants, transition paths and helix boundary diffusion as viewed through the lens of NHB. Except for ALA5, there are definite shifts of population from states with lower NHB to those with higher NHB, related to the helix stabilization. The microscopic reaction rate constants for hydrogen bond transitions are systematically lower in 2 M proline relative to water, reflecting slower overall internal dynamics. The slowing down of peptide conformational transitions in proline has been attributed primarily to the increased solution viscosity, with some effects of peptide compaction and dehydration [18]. In the case of ALA5, the slowing down of folding and unfolding are the main effect, while more changes are found for the longer peptides.

The Global Maximum Weight Paths describe folding transitions following maximum reactive flux [29]. Here, results differ from aqueous simulation, with proline solution bottlenecks shifted toward earlier stages of folding in ALA8 and ALA21, and toward later stages in ALA15. In ODR coarse-grained kinetic models, the direct coil→helix folding mechanism is found for ALA5, ALA8 and ALA15 with proline, but only for the first two peptides in water. For ALA21, the presence of proline greatly simplifies the folding mechanism, which becomes a simple linear folding along a single path, with systematic increase in hydrogen bond count. The transition states for helix folding for the kinetic models shift toward lower NHB values (earlier stages of folding) relative to water. Helix boundary diffusion is also found to be slower in 2 M proline than in water alone. The strongest influence of proline presence is seen in the case of ALA15—this peptide has the largest increases of helix content, slowing of folding relaxation and helix boundary diffusion. Thus, it appears that at this peptide length there is a special effect of proline, with very strong helix stabilization leading to large changes in the folding mechanism.

The resulting picture of helix folding along the NHB coordinate in the presence of proline is subtly different from the more standard results of structure-based kinetic modeling. Overall, the simulations described here present a new way to analyze helix folding and its perturbation by addition of proline as a cosolvent. This helps improve our understanding of osmolyte effects on biological processes and helix folding in general. The folding landscapes of the homogeneous ALAn model systems are only an introduction to the folding of biologically relevant peptides, which may be expected to be more complex.

## Figures and Tables

**Table 1:** Short and long time scales of peptide dynamics in 2 M proline solution. MD—results of two-exponential fits to ACFs [18]. RMSD—kinetic models based on structural clustering [18]. NHB—based on hydrogen bond count coordinate, this work. Details below and in SI (Tables S6a–d and S7a–d). Units: ns.

System	MD C(t) [18]		RMSD [18]		NHB	
	$\tau_{1}$	$\tau_{2}$	$\tau_{1}$	$\tau_{2}$	$\tau_{1}$	$\tau_{2}$
ALA5	~0.2	3	1.6	3.6	2.0	3.3
ALA8	~2	34	2.0	33	8.0	33
ALA15	~20	540	118	520	100	370
ALA21	~20	460	139	470	80	470

**Table 2:** Transition rates between ALA5 hydrogen bonding microstates. $k_{ij}$ is the transition rate for j→i, i,j=0,1,2,3. Diagonal elements are defined as $k_{ii}=-\sum_{j\neq i}k_{ij}\;$ due to probability conservation. State 0 corresponds to the coil, with NHB = 0, state 3 to the helix, with NHB = 3. Units: ${\mu s}^{-1}$.

*i*/*j*	0	1	2	3
0	−21.8	374.9	245.7	206.9
1	16.2	−431.0	93.1	101.3
2	3.0	26.7	−424.2	84.3
3	2.6	29.4	85.4	−392.5

**Table 3:** Transition rates between ALA8 microstates. $k_{ij}$. Notation as in Table 2. State 0 corresponds to the coil, with NHB = 0, state 6 to the helix, with NHB = 6. Units: ${\mu s}^{-1}$.

*i*/*j*	0	1	2	3	4	5	6
0	−13.1	92.8	65.5	43.0	21.6	8.8	7.1
1	7.2	−114.6	13.5	11.7	8.0	4.9	4.2
2	2.9	7.7	−109.4	13.8	9.8	6.3	5.7
3	1.7	5.8	12.0	−103.9	15.1	10.8	10.5
4	0.8	3.6	7.7	13.7	−101.5	21.0	20.8
5	0.3	2.1	4.6	9.1	19.4	−95.5	30.5
6	0.3	2.6	6.0	12.7	27.6	43.7	−78.9

**Table 4:** Properties of two-state ODR models (N = 2). Time constants for folding are inverses of the R-matrix off-diagonal elements in ns. The folding free energy ∆G = G(helix) − G(coil) is calculated from the populations of the aggregate states (ODR). Analogous quantities obtained directly from MD h-bond helix fraction (MD) at 300 K, using a two-state model (ref. [18], details in SI, Tables S7a–d).

System	ODR			MD		
	$\tau_{u}$, ns	$\tau_{f}$, ns	∆G, kcal/mol	$\tau_{u}$, ns	$\tau_{f}$, ns	∆G, kcal/mol
ALA5	3.2	130	2.2	3.8 ± 0.6	35 ± 5	2.01 ± 0.10
ALA8	33	330	0.9	41 ± 10	210 ± 60	1.22 ± 0.04
ALA15	900	550	−0.3	1050 ± 270	1150 ± 360	0.03 ± 0.06
ALA21	3600	470	−1.2	1400 ± 330	710 ± 240	−0.45 ± 0.05

**Table 5:** Hydrogen bond patterns for ALA5, with 3 maximum possible h-bonds. Populations are given as fraction of all structures. NHB denotes the number of formed hydrogen bonds.

Pattern	Population	NHB
000	0.934696	0
001	0.018711	1
100	0.016182	1
010	0.006217	1
011	0.005538	2
110	0.005094	2
101	0.000923	2
111	0.012640	3

**Table 6:** Selected hydrogen bond patterns for ALA15, with 13 maximum possible h-bonds. Notation as in Table 5. More data in Table S9c.

Pattern	Population	NHB
0000000000000	0.252415	0
0000001000000	0.004938	1
0000000000001	0.004704	1
1000000000000	0.004685	1
0000000001000	0.003824	1
0001000000000	0.003456	1
0000100000000	0.003210	1
0000010000000	0.003139	1
0000000010000	0.002747	1
0010000000000	0.002557	1
0000000000010	0.002550	1
…		
0000001111111	0.012893	7
1111111000000	0.011977	7
0001111111000	0.006355	7
0000111111100	0.004585	7
…		
1111111111110	0.035874	12
0111111111111	0.028596	12
1111111111101	0.003089	12
1011111111111	0.002312	12
1111111111011	0.001822	12
1101111111111	0.001099	12
1111111111111	0.116499	13

## Data Availability

The original contributions presented in the study are included in the article/Supplementary Materials, further inquiries can be directed to the corresponding author.

## Supplementary Materials

The following supporting information can be downloaded at: https://www.mdpi.com/article/10.3390/life15050809/s1, Table S1. Hydrogen bond dynamical time scales; Table S2. Ala5 hydrogen bond kinetics; Table S3. Ala8 hydrogen bond kinetics; Table S4. Ala15 hydrogen bond kinetics; Table S5. Ala21 hydrogen bond kinetics; Table S6. Comparison of results from full K matrix and ODR kinetics. (a) ALA5 (b) ALA8 (c) ALA15 (d) ALA21; Table S7. ODR model details. (a) ALA5 (b) ALA8 (c) ALA15 (d) ALA21; Table S8. TPT Committor values (a) ALA5 (b) ALA8 (c) ALA15 (d) ALA21; Table S9. Helical hydrogen bond patterns. (a) ALA5 (b) ALA8 (c) ALA15 (d) ALA21; Table S10. System composition for all MD simulations. Figure S1. Diffusion and friction for helix propagation. References [18,19,25,26,27,28,30,36] are cited in the Supplementary Materials.
