# Supplementary material for: Helix Folding in One Dimension: Effects of Proline Co-Solvent on Free Energy Landscape of Hydrogen Bond Dynamics in Alanine Peptides
Source: Life (Basel). 2025 May 19;15(5):809. doi: 10.3390/life15050809 (PMC12113030; doi:10.3390/life15050809)
Supplement: Supplementary file 1 [file life-15-00809-s001.zip › life-3630678-supplementary.pdf]

# Supplementary Materials

## Helix folding in one dimension: Effects of proline co-solvent on free energy landscape of hydrogen bond dynamics in alanine peptides

Krzysztof Kuczera

**Contents** **Page**

**MD simulation details** … 1

**Optimal Dimensionality Reduction** … 5

**Transition Path Theory** …14

**Hydrogen bond pattern details** …16

**Diffusion and Friction** …23

**System composition (Table S10)** …24

**References** …24

**MD trajectory lengths:**
ALA21 – 5 x 20 $\mu$s
ALA15 – 5 x 10 $\mu$s
ALA8 – 2 x 10 $\mu$s
ALA5 – 2 x 5 $\mu$s

Autocorrelation functions for various quantities x(t) were calculated as

$$C(t) = \langle \Delta x(t) \Delta x(0) \rangle / \langle \Delta x(0)^2 \rangle \text{ with } \Delta x(t) = x(t) - \langle x \rangle$$

Time scales were extracted from ACFs by fitting to a two-exponential decay

$$C(t) = a_0 e^{-t/\tau_1} + (1 - a_0) e^{-t/\tau_2}$$

The slower relaxation times $\tau_2$ were used to a) obtain global folding relaxation times and b) calibrate ODR kinetic simulations. ALA5 and ALA8: a and e denote independent MD trajectories initiated at helix and extended structure, respectively. ALA15 and ALA21, a-e denote five independent MD trajectories initiated at different structures.

Table S1. Hydrogen bond dynamical time scales: individual hydrogen bond length fluctuation ACFs are calculated and averaged, with the average fitted to two-exponential decay.

| ALA5 | a | e |
|---|---|---|

| | $a_0$ | $\tau_1$, ns | $\tau_2$, ns | $a_0$ | $\tau_1$, ns | $\tau_2$,ns |
|---|---|---|---|---|---|---|
| HBAVE | 0.28 | 0.2 | 2.9 | 0.32 | 0.2 | 2.7 |

| ALA8 | h | | | e | | |
|---|---|---|---|---|---|---|
| | $a_0$ | $\tau_1$, ns | $\tau_2$, ns | $a_0$ | $\tau_1$, ns | $\tau_2$,ns |
| HBAVE | 0.29 | 0.90 | 24.1 | 0.39 | 1.6 | 40.0 |

| ALA15 | a | | | b | | | c | | |
|---|---|---|---|---|---|---|---|---|---|
| | $a_0$ | $\tau_1$, ns | $\tau_2$, ns | $a_0$ | $\tau_1$, ns | $\tau_2$,ns | $a_0$ | $\tau_1$, ns | $\tau_2$, ns |
| HBAVE | 0.71 | 63. | 1145 | 0.46 | 36. | 645 | 0.20 | 3.4 | 350 |

| ALA15 | d | | | e | | |
|---|---|---|---|---|---|---|
| | $a_0$ | $\tau_1$, ns | $\tau_2$, ns | $a_0$ | $\tau_1$, ns | $\tau_2$,ns |
| HBAVE | 0.21 | 4.8 | 191 | 0.15 | 2.4 | 169 |

| ALA21 | a | | | b | | | c | | |
|---|---|---|---|---|---|---|---|---|---|
| | $a_0$ | $\tau_1$, ns | $\tau_2$, ns | $a_0$ | $\tau_1$, ns | $\tau_2$,ns | $a_0$ | $\tau_1$, ns | $\tau_2$, ns |
| HBAVE | 0.22 | 8.9 | 210 | 0.40 | 41 | 518 | 0.24 | 8.8 | 317 |

| ALA21 | d | | | e | | |
|---|---|---|---|---|---|---|
| | $a_0$ | $\tau_1$, ns | $\tau_2$, ns | $a_0$ | $\tau_1$, ns | $\tau_2$,ns |
| HBAVE | 0.32 | 29 | 448 | 0.15 | 4.2 | 341 |

Table S2. Ala5 hydrogen bond kinetics. Microstates labelled by number of formed helical hydrogen bonds. Lagtime = 2.0 ns (MD $\tau_2 = 2.8\ ns$ , slowest K matrix relaxation 3.3 ns)

```
...Microstate populations (fractional)
  0.00  0.93578
  1.00  0.04022
  2.00  0.01198
  3.00  0.01202
...Symmetrized transition count matrix T
    0.0   151.5    28.5    24.3
  151.5     0.0    10.8    11.9
   28.5    10.8     0.0     9.9
   24.3    11.9     9.9     0.0
 ... Rates Kij per microsecond
  1    -21.8   374.9   245.7   206.9
  2     16.2  -431.0    93.1   101.3
  3      3.0    26.7  -424.2    84.3
  4      2.6    29.4    85.4  -392.5
```

Table S3. Ala8 hydrogen bond kinetics. Microstates labelled by number of formed helical hydrogen bonds. Lagtime = 8.0 ns (MD $\tau_2 = 32\ ns$ , slowest K matrix relaxation 33 ns)

...Microstate populations (fractional)

| | |
|---|---|
| 0.00 | 0.78516 |
| 1.00 | 0.05864 |
| 2.00 | 0.03566 |
| 3.00 | 0.02979 |
| 4.00 | 0.02743 |
| 5.00 | 0.02498 |
| 6.00 | 0.03834 |

... Symmetrized transition count matrix T

| | | | | | | |
|---|---|---|---|---|---|---|
| 0.0 | 112.7 | 45.7 | 26.1 | 11.9 | 4.5 | 5.2 |
| 112.7 | 0.0 | 9.4 | 7.1 | 4.4 | 2.5 | 3.1 |
| 45.7 | 9.4 | 0.0 | 8.4 | 5.4 | 3.2 | 4.2 |
| 26.1 | 7.1 | 8.4 | 0.0 | 8.3 | 5.5 | 7.7 |
| 11.9 | 4.4 | 5.4 | 8.3 | 0.0 | 10.7 | 15.2 |
| 4.5 | 2.5 | 3.2 | 5.5 | 10.7 | 0.0 | 22.3 |
| 5.2 | 3.1 | 4.2 | 7.7 | 15.2 | 22.3 | 0.0 |

... Rates Kij per microsecond

| | | | | | | | |
|---|---|---|---|---|---|---|---|
| 1 | -13.1 | 92.8 | 65.5 | 43.0 | 21.6 | 8.8 | 7.1 |
| 2 | 7.2 | -114.6 | 13.5 | 11.7 | 8.0 | 4.9 | 4.2 |
| 3 | 2.9 | 7.7 | -109.4 | 13.8 | 9.8 | 6.3 | 5.7 |
| 4 | 1.7 | 5.8 | 12.0 | -103.9 | 15.1 | 10.8 | 10.5 |
| 5 | 0.8 | 3.6 | 7.7 | 13.7 | -101.5 | 21.0 | 20.8 |
| 6 | 0.3 | 2.1 | 4.6 | 9.1 | 19.4 | -95.5 | 30.5 |
| 7 | 0.3 | 2.6 | 6.0 | 12.7 | 27.6 | 43.7 | -78.9 |

Table S4. Ala15 hydrogen bond kinetics. Microstates labelled by number of formed helical hydrogen bonds. Lagtime = 100 ns (MD $\tau_2 = 368\ ns$ , slowest K matrix relaxation 471 ns)

...Microstate populations (fractional)

| | |
|---|---|
| 0 | 0.254217 |
| 1 | 0.043003 |
| 2 | 0.026869 |
| 3 | 0.023700 |
| 4 | 0.030156 |
| 5 | 0.039672 |
| 6 | 0.049284 |
| 7 | 0.058442 |
| 8 | 0.063183 |
| 9 | 0.068981 |
| 10 | 0.078655 |
| 11 | 0.084530 |
| 12 | 0.085383 |
| 13 | 0.093924 |

... Symmetrized transition count matrix T

| | | | | | | | | | | | | | |
|---|---|---|---|---|---|---|---|---|---|---|---|---|---|
| 0.0 | 11.9 | 6.8 | 5.2 | 4.8 | 4.6 | 4.1 | 3.7 | 2.8 | 1.8 | 1.2 | 0.9 | 0.7 | 0.7 |
| 11.9 | 0.0 | 1.1 | 0.9 | 1.0 | 0.9 | 0.9 | 0.8 | 0.6 | 0.4 | 0.4 | 0.2 | 0.2 | 0.2 |
| 6.8 | 1.1 | 0.0 | 0.5 | 0.6 | 0.6 | 0.7 | 0.7 | 0.5 | 0.3 | 0.3 | 0.2 | 0.2 | 0.2 |
| 5.2 | 0.9 | 0.5 | 0.0 | 0.5 | 0.6 | 0.7 | 0.7 | 0.5 | 0.4 | 0.4 | 0.4 | 0.3 | 0.4 |
| 4.8 | 1.0 | 0.6 | 0.5 | 0.0 | 1.0 | 1.1 | 1.0 | 0.9 | 0.7 | 0.8 | 0.7 | 0.6 | 0.7 |
| 4.6 | 0.9 | 0.6 | 0.6 | 1.0 | 0.0 | 1.6 | 1.7 | 1.5 | 1.2 | 1.3 | 1.1 | 1.1 | 1.3 |
| 4.1 | 0.9 | 0.7 | 0.7 | 1.1 | 1.6 | 0.0 | 2.3 | 2.2 | 1.8 | 1.8 | 1.8 | 1.8 | 2.0 |
| 3.7 | 0.8 | 0.7 | 0.7 | 1.0 | 1.7 | 2.3 | 0.0 | 2.7 | 2.3 | 2.4 | 2.6 | 2.7 | 3.0 |

| | | | | | | | | | | | | | |
|---|---|---|---|---|---|---|---|---|---|---|---|---|---|
| 2.8 | 0.6 | 0.5 | 0.5 | 0.9 | 1.5 | 2.2 | 2.7 | 0.0 | 2.8 | 3.3 | 3.4 | 3.6 | 4.0 |
| 1.8 | 0.4 | 0.3 | 0.4 | 0.7 | 1.2 | 1.8 | 2.3 | 2.8 | 0.0 | 4.3 | 4.6 | 4.9 | 5.4 |
| 1.2 | 0.4 | 0.3 | 0.4 | 0.8 | 1.3 | 1.8 | 2.4 | 3.3 | 4.3 | 0.0 | 5.7 | 6.0 | 6.6 |
| 0.9 | 0.2 | 0.2 | 0.4 | 0.7 | 1.1 | 1.8 | 2.6 | 3.4 | 4.6 | 5.7 | 0.0 | 6.8 | 7.5 |
| 0.7 | 0.2 | 0.2 | 0.3 | 0.6 | 1.1 | 1.8 | 2.7 | 3.6 | 4.9 | 6.0 | 6.8 | 0.0 | 7.3 |
| 0.7 | 0.2 | 0.2 | 0.4 | 0.7 | 1.3 | 2.0 | 3.0 | 4.0 | 5.4 | 6.6 | 7.5 | 7.3 | 0.0 |

... Rates Kij per microsecond

| | | | | | | | | | | | | | | |
|---|---|---|---|---|---|---|---|---|---|---|---|---|---|---|
| 1 | 0.0 | 5.6 | 5.1 | 4.4 | 3.2 | 2.3 | 1.7 | 1.3 | 0.9 | 0.5 | 0.3 | 0.2 | 0.2 | 0.1 |
| 2 | 0.9 | 0.0 | 0.8 | 0.8 | 0.6 | 0.5 | 0.4 | 0.3 | 0.2 | 0.1 | 0.1 | 0.1 | 0.0 | 0.0 |
| 3 | 0.5 | 0.5 | 0.0 | 0.4 | 0.4 | 0.3 | 0.3 | 0.2 | 0.2 | 0.1 | 0.1 | 0.1 | 0.0 | 0.0 |
| 4 | 0.4 | 0.4 | 0.4 | 0.0 | 0.4 | 0.3 | 0.3 | 0.2 | 0.2 | 0.1 | 0.1 | 0.1 | 0.1 | 0.1 |
| 5 | 0.4 | 0.4 | 0.4 | 0.5 | 0.0 | 0.5 | 0.4 | 0.3 | 0.3 | 0.2 | 0.2 | 0.2 | 0.1 | 0.2 |
| 6 | 0.4 | 0.4 | 0.5 | 0.5 | 0.6 | 0.0 | 0.6 | 0.6 | 0.5 | 0.4 | 0.3 | 0.3 | 0.3 | 0.3 |
| 7 | 0.3 | 0.4 | 0.5 | 0.6 | 0.7 | 0.8 | 0.0 | 0.8 | 0.7 | 0.5 | 0.5 | 0.4 | 0.4 | 0.4 |
| 8 | 0.3 | 0.4 | 0.5 | 0.6 | 0.7 | 0.8 | 0.9 | 0.0 | 0.9 | 0.7 | 0.6 | 0.6 | 0.6 | 0.6 |
| 9 | 0.2 | 0.3 | 0.4 | 0.4 | 0.6 | 0.7 | 0.9 | 0.9 | 0.0 | 0.8 | 0.8 | 0.8 | 0.8 | 0.9 |
| 10 | 0.1 | 0.2 | 0.2 | 0.3 | 0.5 | 0.6 | 0.7 | 0.8 | 0.9 | 0.0 | 1.1 | 1.1 | 1.2 | 1.2 |
| 11 | 0.1 | 0.2 | 0.2 | 0.4 | 0.5 | 0.6 | 0.7 | 0.8 | 1.0 | 1.2 | 0.0 | 1.3 | 1.4 | 1.4 |
| 12 | 0.1 | 0.1 | 0.2 | 0.3 | 0.4 | 0.6 | 0.7 | 0.9 | 1.1 | 1.3 | 1.4 | 0.0 | 1.6 | 1.6 |
| 13 | 0.1 | 0.1 | 0.1 | 0.3 | 0.4 | 0.6 | 0.7 | 0.9 | 1.1 | 1.4 | 1.5 | 1.6 | 0.0 | 1.5 |
| 14 | 0.1 | 0.1 | 0.2 | 0.3 | 0.5 | 0.7 | 0.8 | 1.0 | 1.3 | 1.6 | 1.7 | 1.8 | 1.7 | 0.0 |

Table S5. Ala21 hydrogen bond kinetics. Microstates labelled by number of formed helical hydrogen bonds. Lagtime = 80 ns (MD $\tau_2 = 500\ ns$ , slowest K matrix relaxation 367 ns)

...Microstate populations (fractional)

| | |
|---|---|
| 0 | 0.056880 |
| 1 | 0.013524 |
| 2 | 0.007725 |
| 3 | 0.008018 |
| 4 | 0.013742 |
| 5 | 0.015655 |
| 6 | 0.017139 |
| 7 | 0.024338 |
| 8 | 0.028274 |
| 9 | 0.034874 |
| 10 | 0.045709 |
| 11 | 0.057595 |
| 12 | 0.063033 |
| 13 | 0.070752 |
| 14 | 0.076789 |
| 15 | 0.078798 |
| 16 | 0.090573 |
| 17 | 0.098709 |
| 18 | 0.104445 |
| 19 | 0.093429 |

...Symmetrized transition count matrix

| | | | | | | | | | | | | | | | | | | | |
|---|---|---|---|---|---|---|---|---|---|---|---|---|---|---|---|---|---|---|---|
| 0.0 | 8.9 | 4.0 | 2.9 | 3.5 | 3.4 | 2.0 | 1.7 | 1.5 | 1.0 | 0.8 | 0.6 | 0.3 | 0.2 | 0.0 | 0.0 | 0.0 | 0.0 | 0.0 | 0.0 |
| 8.9 | 0.0 | 0.9 | 0.7 | 0.9 | 1.0 | 0.5 | 0.5 | 0.4 | 0.3 | 0.2 | 0.2 | 0.1 | 0.1 | 0.0 | 0.0 | 0.0 | 0.0 | 0.0 | 0.0 |
| 4.0 | 0.9 | 0.0 | 0.4 | 0.6 | 0.7 | 0.5 | 0.5 | 0.4 | 0.2 | 0.2 | 0.2 | 0.1 | 0.1 | 0.1 | 0.0 | 0.0 | 0.0 | 0.0 | 0.0 |
| 2.9 | 0.7 | 0.4 | 0.0 | 0.9 | 0.8 | 0.7 | 0.6 | 0.5 | 0.3 | 0.3 | 0.3 | 0.2 | 0.2 | 0.2 | 0.1 | 0.1 | 0.1 | 0.0 | 0.0 |
| 3.5 | 0.9 | 0.6 | 0.9 | 0.0 | 1.6 | 1.3 | 1.3 | 1.1 | 0.8 | 0.7 | 0.5 | 0.4 | 0.4 | 0.4 | 0.3 | 0.2 | 0.1 | 0.1 | 0.0 |
| 3.4 | 1.0 | 0.7 | 0.8 | 1.6 | 0.0 | 1.5 | 1.5 | 1.5 | 1.2 | 1.0 | 0.9 | 0.7 | 0.6 | 0.5 | 0.3 | 0.2 | 0.1 | 0.1 | 0.1 |
| 2.0 | 0.5 | 0.5 | 0.7 | 1.3 | 1.5 | 0.0 | 2.1 | 1.8 | 1.6 | 1.5 | 1.5 | 1.3 | 1.0 | 0.7 | 0.4 | 0.3 | 0.3 | 0.2 | 0.2 |
| 1.7 | 0.5 | 0.5 | 0.6 | 1.3 | 1.5 | 2.1 | 0.0 | 2.8 | 2.6 | 2.8 | 2.6 | 2.2 | 1.8 | 1.3 | 0.8 | 0.6 | 0.5 | 0.4 | 0.4 |
| 1.5 | 0.4 | 0.4 | 0.5 | 1.1 | 1.5 | 1.8 | 2.8 | 0.0 | 3.5 | 3.6 | 3.6 | 3.1 | 2.6 | 1.9 | 1.1 | 0.8 | 0.7 | 0.7 | 0.6 |
| 1.0 | 0.3 | 0.2 | 0.3 | 0.8 | 1.2 | 1.6 | 2.6 | 3.5 | 0.0 | 5.0 | 5.2 | 4.3 | 3.8 | 2.8 | 1.9 | 1.3 | 1.2 | 1.1 | 1.0 |
| 0.8 | 0.2 | 0.2 | 0.3 | 0.7 | 1.0 | 1.5 | 2.8 | 3.6 | 5.0 | 0.0 | 7.4 | 6.3 | 5.6 | 4.4 | 3.0 | 2.4 | 2.0 | 1.9 | 1.7 |
| 0.6 | 0.2 | 0.2 | 0.3 | 0.5 | 0.9 | 1.5 | 2.6 | 3.6 | 5.2 | 7.4 | 0.0 | 8.7 | 8.1 | 6.7 | 4.2 | 3.3 | 2.9 | 2.9 | 2.6 |
| 0.3 | 0.1 | 0.1 | 0.2 | 0.4 | 0.7 | 1.3 | 2.2 | 3.1 | 4.3 | 6.3 | 8.7 | 0.0 | 9.8 | 8.6 | 5.4 | 4.7 | 4.5 | 4.5 | 4.1 |
| 0.2 | 0.1 | 0.1 | 0.2 | 0.4 | 0.6 | 1.0 | 1.8 | 2.6 | 3.8 | 5.6 | 8.1 | 9.8 | 0.0 | 10.3 | 7.1 | 6.6 | 6.5 | 6.7 | 6.0 |
| 0.0 | 0.0 | 0.1 | 0.2 | 0.4 | 0.5 | 0.7 | 1.3 | 1.9 | 2.8 | 4.4 | 6.7 | 8.6 | 10.3 | 0.0 | 9.1 | 9.6 | 9.7 | 10.0 | 9.0 |
| 0.0 | 0.0 | 0.0 | 0.1 | 0.3 | 0.3 | 0.4 | 0.8 | 1.1 | 1.9 | 3.0 | 4.2 | 5.4 | 7.1 | 9.1 | 0.0 | 12.9 | 13.7 | 14.4 | 12.8 |
| 0.0 | 0.0 | 0.0 | 0.1 | 0.2 | 0.2 | 0.3 | 0.6 | 0.8 | 1.3 | 2.4 | 3.3 | 4.7 | 6.6 | 9.6 | 12.9 | 0.0 | 17.9 | 19.3 | 17.3 |
| 0.0 | 0.0 | 0.0 | 0.1 | 0.1 | 0.1 | 0.3 | 0.5 | 0.7 | 1.2 | 2.0 | 2.9 | 4.5 | 6.5 | 9.7 | 13.7 | 17.9 | 0.0 | 22.7 | 20.4 |
| 0.0 | 0.0 | 0.0 | 0.0 | 0.1 | 0.1 | 0.2 | 0.4 | 0.7 | 1.1 | 1.9 | 2.9 | 4.5 | 6.7 | 10.0 | 14.4 | 19.3 | 22.7 | 0.0 | 21.6 |
| 0.0 | 0.0 | 0.0 | 0.0 | 0.0 | 0.1 | 0.2 | 0.4 | 0.6 | 1.0 | 1.7 | 2.6 | 4.1 | 6.0 | 9.0 | 12.8 | 17.3 | 20.4 | 21.6 | 0.0 |

... Rates Kj per microsecond

| | | | | | | | | | | | | | | | | | | | | |
|---|---|---|---|---|---|---|---|---|---|---|---|---|---|---|---|---|---|---|---|---|
| 1 | -5.4 | 6.6 | 5.2 | 3.6 | 2.5 | 2.2 | 1.2 | 0.7 | 0.5 | 0.3 | 0.2 | 0.1 | 0.0 | 0.0 | 0.0 | 0.0 | 0.0 | 0.0 | 0.0 | 0.0 |
| 2 | 1.6 | -10.9 | 1.2 | 0.9 | 0.7 | 0.6 | 0.3 | 0.2 | 0.1 | 0.1 | 0.0 | 0.0 | 0.0 | 0.0 | 0.0 | 0.0 | 0.0 | 0.0 | 0.0 | 0.0 |
| 3 | 0.7 | 0.7 | -11.5 | 0.5 | 0.4 | 0.4 | 0.3 | 0.2 | 0.1 | 0.1 | 0.0 | 0.0 | 0.0 | 0.0 | 0.0 | 0.0 | 0.0 | 0.0 | 0.0 | 0.0 |
| 4 | 0.5 | 0.5 | 0.5 | -11.6 | 0.7 | 0.5 | 0.4 | 0.2 | 0.2 | 0.1 | 0.1 | 0.1 | 0.0 | 0.0 | 0.0 | 0.0 | 0.0 | 0.0 | 0.0 | 0.0 |
| 5 | 0.6 | 0.7 | 0.8 | 1.1 | -11.0 | 1.0 | 0.8 | 0.5 | 0.4 | 0.2 | 0.2 | 0.1 | 0.1 | 0.1 | 0.1 | 0.0 | 0.0 | 0.0 | 0.0 | 0.0 |
| 6 | 0.6 | 0.7 | 0.9 | 1.0 | 1.2 | -11.3 | 0.9 | 0.6 | 0.5 | 0.3 | 0.2 | 0.2 | 0.1 | 0.1 | 0.1 | 0.0 | 0.0 | 0.0 | 0.0 | 0.0 |
| 7 | 0.4 | 0.4 | 0.6 | 0.9 | 0.9 | 1.0 | -11.3 | 0.9 | 0.6 | 0.5 | 0.3 | 0.3 | 0.2 | 0.1 | 0.1 | 0.1 | 0.0 | 0.0 | 0.0 | 0.0 |
| 8 | 0.3 | 0.4 | 0.6 | 0.7 | 0.9 | 1.0 | 1.2 | -11.1 | 1.0 | 0.7 | 0.6 | 0.5 | 0.3 | 0.3 | 0.2 | 0.1 | 0.1 | 0.1 | 0.0 | 0.0 |
| 9 | 0.3 | 0.3 | 0.5 | 0.6 | 0.8 | 1.0 | 1.1 | 1.2 | -11.4 | 1.0 | 0.8 | 0.6 | 0.5 | 0.4 | 0.2 | 0.1 | 0.1 | 0.1 | 0.1 | 0.1 |
| 10 | 0.2 | 0.2 | 0.3 | 0.4 | 0.6 | 0.8 | 0.9 | 1.1 | 1.2 | -11.2 | 1.1 | 0.9 | 0.7 | 0.5 | 0.4 | 0.2 | 0.1 | 0.1 | 0.1 | 0.1 |

| | | | | | | | | | | | | | | | | | | | | |
|---|---|---|---|---|---|---|---|---|---|---|---|---|---|---|---|---|---|---|---|---|
| 11 | 0.1 | 0.1 | 0.3 | 0.4 | 0.5 | 0.6 | 0.9 | 1.2 | 1.3 | 1.4 | -11.1 | 1.3 | 1.0 | 0.8 | 0.6 | 0.4 | 0.3 | 0.2 | 0.2 | 0.2 |
| 12 | 0.1 | 0.1 | 0.3 | 0.4 | 0.4 | 0.6 | 0.9 | 1.1 | 1.3 | 1.5 | 1.6 | -10.8 | 1.4 | 1.1 | 0.9 | 0.5 | 0.4 | 0.3 | 0.3 | 0.3 |
| 13 | 0.1 | 0.1 | 0.1 | 0.2 | 0.3 | 0.4 | 0.8 | 0.9 | 1.1 | 1.2 | 1.4 | 1.5 | -11.0 | 1.4 | 1.1 | 0.7 | 0.5 | 0.5 | 0.4 | 0.4 |
| 14 | 0.0 | 0.1 | 0.1 | 0.2 | 0.3 | 0.4 | 0.6 | 0.7 | 0.9 | 1.1 | 1.2 | 1.4 | 1.6 | -11.0 | 1.3 | 0.9 | 0.7 | 0.7 | 0.6 | 0.6 |
| 15 | 0.0 | 0.0 | 0.1 | 0.2 | 0.3 | 0.3 | 0.4 | 0.5 | 0.7 | 0.8 | 1.0 | 1.2 | 1.4 | 1.5 | -11.1 | 1.2 | 1.1 | 1.0 | 1.0 | 1.0 |
| 16 | 0.0 | 0.0 | 0.0 | 0.1 | 0.2 | 0.2 | 0.2 | 0.3 | 0.4 | 0.5 | 0.7 | 0.7 | 0.9 | 1.0 | 1.2 | -11.1 | 1.4 | 1.4 | 1.4 | 1.4 |
| 17 | 0.0 | 0.0 | 0.0 | 0.1 | 0.1 | 0.1 | 0.2 | 0.2 | 0.3 | 0.4 | 0.5 | 0.6 | 0.7 | 0.9 | 1.3 | 1.6 | -10.8 | 1.8 | 1.8 | 1.9 |
| 18 | 0.0 | 0.0 | 0.0 | 0.1 | 0.1 | 0.1 | 0.2 | 0.2 | 0.2 | 0.3 | 0.4 | 0.5 | 0.7 | 0.9 | 1.3 | 1.7 | 2.0 | -10.5 | 2.2 | 2.2 |
| 19 | 0.0 | 0.0 | 0.0 | 0.0 | 0.1 | 0.1 | 0.1 | 0.2 | 0.2 | 0.3 | 0.4 | 0.5 | 0.7 | 0.9 | 1.3 | 1.8 | 2.1 | 2.3 | -10.2 | 2.3 |
| 20 | 0.0 | 0.0 | 0.0 | 0.0 | 0.0 | 0.1 | 0.1 | 0.2 | 0.2 | 0.3 | 0.4 | 0.5 | 0.7 | 0.8 | 1.2 | 1.6 | 1.9 | 2.1 | 2.1 | -10.5 |

## Kinetic coarse graining with ODR

Detailed coarse graining protocol.

1. Trajectories are discretized using a smoothed hydrogen bond count NHB(t) (Methods). The microstates are labeled 1,2,…,MAXHB+1, where MAXHB is the maximum possible number of helical hydrogen bonds; MAXHB=3, 6, 13 and 19 for ALA5, ALA8, ALA15 and ALA21, respectively. State 1 corresponds to number of hydrogen bonds NHB=0, state 2 to NHB=1, etc.
2. The residence time $t_i$ in state i is counted simply as the time the value of the smoothed hydrogen bond count NHB(t) was closest to NHB=i-1 (see Methods in main text).
3. Transitions between microstates are counted using the moving window approach with a lagtime $\tau_l$ to smooth out fast structural fluctuations[1]. The value of $\tau_l$ for each system was chosen so that the slowest relaxation time agreed with results from decays of autocorrelation functions of global quantities sampled in MD trajectories. The quantities chosen in this work are: RMSDH(t) – CA atom RMSD from ideal helix, HBCNT(t) – number of helical hydrogen bonds present and HBAVE(t) – length fluctuations of helical hydrogen bonds, averaged over all MAXHB hydrogen bonds. The transition matrix was symmetrized $T_{ij} = (T_{ij}^* + T_{ji}^*)$ to obtain the best estimate. The kinetic rate matrix is calculated as $K_{ij} = T_{ij}/t_j$ [2].
4. Microstates are divided into a small number N of aggregate states, sometimes called metastable states in the literature, using the PCCA+[3] algorithm implemented in the package EMMA 1.4[4]. This algorithm groups states according to the sign structure of the eigenvectors of the Transition matrix $T_{ij}$, which are the same as the eigenvectors of the kinetic rate matrix $K_{ij}$.
5. Using a small number of aggregate states, N=2-5, we use the Optimal Dimensionality Reduction method to generate an effective rate matrix **R** which best represents the slowest dynamical processes in the system.[5] The NxN matrix **R** is based on the full $N_c$x$N_c$ rate matrix **K** and the aggregate state definitions as follows:

$$\boldsymbol{R} = \boldsymbol{P}_{eq}\boldsymbol{1}_N^T - \boldsymbol{D}_N\left[\boldsymbol{A}^T\left(\boldsymbol{p}_{eq}\boldsymbol{1}_n^T - \boldsymbol{K}\right)^{-1}\boldsymbol{D}_n\boldsymbol{A}\right]^{-1}$$

Here $\mathbf{1}_n$ and $\mathbf{1}_N$ are the unit matrices of the higher and lower dimensional space, respectively, $\mathbf{p}_{eq}$ and $\mathbf{P}_{eq}$ are the corresponding diagonal matrices with equilibrium populations, $\mathbf{D}_n$ and $\mathbf{D}_N$ the diagonal matrices of eigenvalues. **K** is the n x n kinetic matrix and **A** is the n x N transformation matrix from higher to lower dimensional space, such that $\mathbf{P}_{eq} = \mathbf{A}^T\mathbf{p}_{eq}$, with T denoting a matrix transpose.[5]

Definitions of quantities presented below:

Aggregate rate matrices **R** and aggregate state properties calculated for N=2-5 aggregate states. Values marked in red are considered too low to be reliable, given the trajectory lengths, and are ignored in analysis, i.e. rates are effectively set to zero.

Off-diagonal elements $R_{ij}$ are rates for j to i transitions, while the diagonal element $-1/R_{ii}$ is the lifetime of state i. $R_{ij}$ values are given in $ns^{-1}$ and $microseconds^{-1}$ .

Aggregate state properties are:
NHB – number of alpha-helical h-bonds (between C=O of residue i and N-H of residue i+4, including blocking groups, averaged over constituent MD frames;
RMSD – CA atom RMSD from ideal helix, averaged over constituent MD frames, A;
population – fraction of trajectory time spent in state i;

$\Delta G$ – free energy relative to most populated state (kcal/mol), at T=300 K

$\Delta G_i = -RT \ln\left(\frac{pop_i}{pop_{max}}\right)$; R = 8.314 J $mol^{-1}$ $K^{-1}$

Ns – number of original microstates contributing to this aggregate state;
List – list of contributing microstates, e.g. [1,2]=x means this aggregate is made up of microstates 1 and 2 (i.e. NHB=0 and NHB=1) and corresponds to the coil (x=c), helix (x=h) or intermediate [x=i].

**Ala5. Number of microstates $N_c$ = 4, lagtime $\tau_l = 2.0\ ns$**

Table S6a.
Comparison of results from full K matrix (dimension $N_c$ = 4) and reduced dimensionality models with dimensions N=2-3. Relaxation times, ns.

| Full K matrix | N=2 | N=3 | N=4* |
|---|---|---|---|
| 3.33 | 3.11 | 3.32 | 3.33 |
| 2.10 | | 2.10 | 2.10 |
| 2.02 | | | 2.02 |

*N=4 corresponds to full microstate space

Table S7a. ODR models for ALA5
N=2
... Lifetimes -1/EV(I) [ns], I=1,NS-1
3.113461
... Aggregate rates RHS [ns-1]
1 -0.007498 0.313688
2 0.007498 -0.313688
... Aggregate rates RHS [microseconds-1]
1 -7.498 313.688
2 7.498 -313.688

... Times=1/rates [ns] with RCUT=  0.000200

| | | |
|---|---|---|
| 1 | 133.37 | 3.19 |
| 2 | 133.37 | 3.19 |

Properties

| # | NHB | RMSDH | Pop | $\Delta G, kcal/mol$ | Ns | List |
|---|---|---|---|---|---|---|
| 1 | 0.0 | 2.7 | 0.9766270 | 0.0000 | 2 | [1-2] |
| 2 | 2.5 | 0.2 | 0.0233730 | 2.2251 | 2 | [3-4] |

N=3

... Lifetimes -1/EV(I) [ns], I=1,NS-1

2.103229

3.324729

... Aggregate rates RHS [ns-1]

| | | | |
|---|---|---|---|
| 1 | -0.431164 | 0.097485 | 0.016180 |
| 2 | 0.056313 | -0.323262 | 0.005630 |
| 3 | 0.374850 | 0.225777 | -0.021810 |

... Aggregate rates RHS [microseconds-1]

| | | | |
|---|---|---|---|
| 1 | -431.164 | 97.485 | 16.180 |
| 2 | 56.313 | -323.262 | 5.630 |
| 3 | 374.850 | 225.777 | -21.810 |

... Times=1/rates [ns] with RCUT=  0.000200

| | | | |
|---|---|---|---|
| 1 | 2.32 | 10.26 | 61.80 |
| 2 | 17.76 | 3.09 | 177.63 |
| 3 | 2.67 | 4.43 | 45.85 |

Properties

| # | NHB | RMSDH | Pop | $\Delta G, kcal/mol$ | Ns | List |
|---|---|---|---|---|---|---|
| 1 | 1.0 | 1.5 | 0.0404840 | 1.8724 | 1 | [2] |
| 2 | 2.5 | 0.2 | 0.0233730 | 2.1998 | 2 | [3-4] |
| 3 | 0.0 | 2.8 | 0.9361431 | 0.0000 | 1 | [1] |

N=4

... Lifetimes -1/EV(I) [ns], I=1,NS-1

2.017390

2.105178

3.333009

... Aggregate rates RHS [microseconds-1]

| | | | | |
|---|---|---|---|---|
| 1 | -21.829 | 374.942 | 245.701 | 207.274 |
| 2 | 16.184 | -431.183 | 93.257 | 101.412 |
| 3 | 3.044 | 26.765 | -424.539 | 84.502 |
| 4 | 2.601 | 29.476 | 85.580 | -393.188 |

... Times=1/rates [ns] with RCUT=  0.000200

| | | | | |
|---|---|---|---|---|
| 1 | 45.81 | 2.67 | 4.07 | 4.82 |
| 2 | 61.79 | 2.32 | 10.72 | 9.86 |
| 3 | 328.54 | 37.36 | 2.36 | 11.83 |
| 4 | 384.54 | 33.93 | 11.68 | 2.54 |

Properties

| # | NHB | RMSDH | Pop | $\Delta G, kcal/mol$ | Ns | List |
|---|---|---|---|---|---|---|
| 1 | 0.0 | 2.8 | 0.9361431 | 0.0000 | 1 | [1] |

| | | | | | | |
|---|---|---|---|---|---|---|
| 2 | 1.0 | 1.5 | 0.0404840 | 1.8724 | 1 | [2] |
| 3 | 2.0 | 0.3 | 0.0116680 | 2.6140 | 1 | [3] |
| 4 | 3.0 | 0.2 | 0.0117050 | 2.6121 | 1 | [4] |

**Ala8. Number of microstates $N_c$ = 7, lagtime $\tau_l = 8.0\ ns$**

Table S6b.

Comparison of results from full K matrix (dimension $N_c$ = 7) and reduced dimensionality models with dimensions N=2-5. Relaxation times, ns.

| Full K matrix | N=2 | N=3 | N=4 | N=5 |
|---|---|---|---|---|
| 32.7 | 29.7 | 31.2 | 32.0 | 32.3 |
| 10.2 | | 9.8 | 9.8 | 9.8 |
| 8.4 | | | 8.2 | 8.3 |
| 8.3 | | | | 8.2 |

Table S7b. ODR models for ALA8

N=2

... Lifetimes -1/EV(I) [ns], I=1,NS-1

29.733624

... Aggregate rates RHS [ns-1]

1 -0.003013 0.030618

2 0.003013 -0.030618

... Aggregate rates RHS [microseconds-1]

1 -3.013 30.618

2 3.013 -30.618

... Times=1/rates [ns] with RCUT= 0.000100

1 331.84 32.66

2 331.84 32.66

Properties

| # | NHB | RMSDH | Pop | $\Delta G, kcal/mol$ | Ns | List |
|---|---|---|---|---|---|---|
| 1 | 0.2 | 3.8 | 0.9103990 | 0.0000 | 4 | [1-4] |
| 2 | 5.1 | 0.5 | 0.0896010 | 1.3821 | 3 | [5-7] |

N=3

... Lifetimes -1/EV(I) [ns], I=1,NS-1

9.827323

31.182083

... Aggregate rates RHS [ns-1]

| | | | |
|---|---|---|---|
| 1 | -0.080482 | 0.038779 | 0.005985 |
| 2 | 0.025950 | -0.046773 | 0.000587 |
| 3 | 0.054532 | 0.007994 | -0.006572 |

... Aggregate rates RHS [microseconds-1]

| | | | |
|---|---|---|---|
| 1 | -80.482 | 38.779 | 5.985 |
| 2 | 25.950 | -46.773 | 0.587 |
| 3 | 54.532 | 7.994 | -6.572 |

... Times=1/rates [ns] with RCUT= 0.000100

| | | | |
|---|---|---|---|
| 1 | 12.43 | 25.79 | 167.09 |
| 2 | 38.54 | 21.38 | 1703.15 |
| 3 | 18.34 | 125.09 | 152.16 |

Properties

| # | NHB | RMSDH | Pop | $\Delta G, kcal/mol$ | Ns | List |
|---|---|---|---|---|---|---|
| 1 | 2.9 | 2.1 | 0.0928444 | 1.3166 | 3 | [3-5] |
| 2 | 5.6 | 0.2 | 0.0620590 | 1.5567 | 2 | [6-7] |
| 3 | 0.1 | 3.9 | 0.8450966 | 0.0000 | 2 | [1-2] |

N=4

... Lifetimes -1/EV(I) [ns], I=1,NS-1

8.171023

9.762360

32.044046

... Aggregate rates RHS [ns-1]

| | | | | |
|---|---|---|---|---|
| 1 | -0.078030 | 0.052097 | 0.017538 | 0.001288 |
| 2 | 0.035932 | -0.077916 | 0.009151 | 0.000411 |
| 3 | 0.021574 | 0.016322 | -0.093244 | 0.005137 |
| 4 | 0.020524 | 0.009497 | 0.066554 | -0.006835 |

... Aggregate rates RHS [microseconds-1]

| | | | | |
|---|---|---|---|---|
| 1 | -78.030 | 52.097 | 17.538 | 1.288 |
| 2 | 35.932 | -77.916 | 9.151 | 0.411 |
| 3 | 21.574 | 16.322 | -93.244 | 5.137 |
| 4 | 20.524 | 9.497 | 66.554 | -6.835 |

... Times=1/rates [ns] with RCUT= 0.000100

| | | | | |
|---|---|---|---|---|
| 1 | 12.82 | 19.19 | 57.02 | 776.55 |
| 2 | 27.83 | 12.83 | 109.27 | 2433.37 |
| 3 | 46.35 | 61.27 | 10.72 | 194.68 |
| 4 | 48.72 | 105.30 | 15.03 | 146.30 |

Properties

| # | NHB | RMSDH | Pop | $\Delta G, kcal/mol$ | Ns | List |
|---|---|---|---|---|---|---|
| 1 | 4.5 | 0.8 | 0.0531490 | 1.6491 | 2 | [5-6] |
| 2 | 6.0 | 0.2 | 0.0364520 | 1.8739 | 1 | [7] |
| 3 | 2.5 | 2.5 | 0.0653025 | 1.5263 | 2 | [3-4] |
| 4 | 0.1 | 3.9 | 0.8450966 | 0.0000 | 2 | [1-2] |

N=5

... Lifetimes -1/EV(I) [ns], I=1,NS-1

```
   8.164594
   8.304324
   9.781928
  32.263141
 ... Aggregate rates RHS [ns-1]
  1 -0.078302  0.051845  0.022887  0.012582  0.001328
  2  0.035757 -0.078077  0.012574  0.005980  0.000437
  3  0.013107  0.010440 -0.103624  0.012387  0.001938
  4  0.008272  0.005701  0.014221 -0.109190  0.003227
  5  0.021166  0.010092  0.053942  0.078241 -0.006930
 ... Aggregate rates RHS [microseconds-1]
  1   -78.302   51.845   22.887   12.582    1.328
  2    35.757  -78.077   12.574    5.980    0.437
  3    13.107   10.440 -103.624   12.387    1.938
  4     8.272    5.701   14.221 -109.190    3.227
  5    21.166   10.092   53.942   78.241   -6.930
... Times=1/rates [ns] with RCUT=  0.000100
  1      12.77      19.29      43.69      79.48     753.01
  2      27.97      12.81      79.53     167.21    2289.86
  3      76.30      95.79       9.65      80.73     515.95
  4     120.89     175.42      70.32       9.16     309.84
  5      47.25      99.09      18.54      12.78     144.29
Properties
  #    NHB    RMSDH Pop      ΔG, kcal/mol  Ns   List
  1    4.5     0.8  0.0531490    1.6491   2    [5-6]
  2    6.0     0.2  0.0364520    1.8739   1    [7]
  3    3.0     2.2  0.0304305    1.9815   1    [4]
  4    2.0     2.8  0.0348720    1.9003   1    [3]
  5    0.1     3.9  0.8450966    0.0000   2    [1-2]
```

### Ala15. Number of microstates $N_c$ = 14, lagtime $\tau_l = 100\ ns$

Table S6c.
Comparison of results from full K matrix (dimension $N_c$ = 11) and reduced dimensionality models with dimensions N=2-5. Relaxation times, ns.

| Full K matrix | N=2 | N=3 | N=4 | N=5 |
|---|---|---|---|---|
| 368 | 340 | 358 | 363 | 364 |
| 117 | | 115 | 115 | 115 |
| 102 | | | 101 | 101 |
| 100 | | | | 98 |
| 100 | | | | |

Table S7c. ODR models for ALA15

N=2

... Lifetimes -1/EV(I) [ns], I=1,NS-1

339.526911

... Aggregate rates RHS [ns-1]

| | | |
|---|---|---|
| 1 | -0.001832 | 0.001113 |
| 2 | 0.001832 | -0.001113 |

... Aggregate rates RHS [microseconds-1]

| | | |
|---|---|---|
| 1 | -1.832 | 1.113 |
| 2 | 1.832 | -1.113 |

... Times=1/rates [ns] with RCUT= 0.000100

| | | |
|---|---|---|
| 1 | 545.82 | 898.35 |
| 2 | 545.82 | 898.35 |

Properties

| # | NHB | RMSDH | Pop | $\Delta G, kcal/mol$ | Ns | List |
|---|---|---|---|---|---|---|
| 1 | 0.8 | 5.8 | 0.3779049 | 0.2971 | 5 | [1-5] |
| 2 | 9.7 | 2.0 | 0.6220951 | 0.0000 | 9 | [6-14] |

N=3

... Lifetimes -1/EV(I) [ns], I=1,NS-1

114.888290
358.104072

... Aggregate rates RHS [ns-1]

| | | | |
|---|---|---|---|
| 1 | -0.002186 | 0.002500 | 0.000385 |
| 2 | 0.001731 | -0.006554 | 0.002372 |
| 3 | 0.000456 | 0.004054 | -0.002757 |

... Aggregate rates RHS [microseconds-1]

| | | | |
|---|---|---|---|
| 1 | -2.186 | 2.500 | 0.385 |
| 2 | 1.731 | -6.554 | 2.372 |
| 3 | 0.456 | 4.054 | -2.757 |

... Times=1/rates [ns] with RCUT= 0.000100

| | | | |
|---|---|---|---|
| 1 | 457.40 | 399.99 | 2596.23 |
| 2 | 577.85 | 152.59 | 421.66 |
| 3 | 2194.41 | 246.70 | 362.75 |

Properties

| # | NHB | RMSDH | Pop | $\Delta G, kcal/mol$ | Ns | List |
|---|---|---|---|---|---|---|
| 1 | 0.5 | 5.9 | 0.3477381 | 0.1002 | 4 | [1-4] |
| 2 | 6.5 | 3.7 | 0.2408791 | 0.3191 | 5 | [5-9] |
| 3 | 11.2 | 1.1 | 0.4113827 | 0.0000 | 5 | [10-14] |

N=4

... Lifetimes -1/EV(I) [ns], I=1,NS-1

101.423139
114.925437
363.316874

... Aggregate rates RHS [ns-1]

| | | | | |
|---|---|---|---|---|
| 1 | -0.008838 | 0.000905 | 0.000785 | 0.000460 |
| 2 | 0.002216 | -0.007431 | 0.000948 | 0.001909 |
| 3 | 0.003910 | 0.001930 | -0.002244 | 0.000431 |

```
  4  0.002712  0.004596  0.000510 -0.002801
 ... Aggregate rates RHS [microseconds-1]
   1    -8.838     0.905     0.785     0.460
   2     2.216    -7.431     0.948     1.909
   3     3.910     1.930    -2.244     0.431
   4     2.712     4.596     0.510    -2.801
... Times=1/rates [ns] with RCUT=  0.000100
   1      113.15     1104.61     1273.93     2172.72
   2      451.31      134.57     1054.46      523.81
   3      255.78      518.18      445.68     2317.86
   4      368.72      217.57     1959.12      357.05
```

Properties

| # | NHB | RMSDH | Pop | $\Delta G, kcal/mol$ | Ns | List |
|---|---|---|---|---|---|---|
| 1 | 4.7 | 4.5 | 0.0698900 | 1.0567 | 2 | [5-6] |
| 2 | 7.2 | 3.4 | 0.1709892 | 0.5234 | 3 | [7-9] |
| 3 | 0.5 | 5.9 | 0.3477381 | 0.1002 | 4 | [1-4] |
| 4 | 11.2 | 1.1 | 0.4113827 | 0.0000 | 5 | [10-14] |

N=5

```
... Lifetimes -1/EV(I) [ns], I=1,NS-1
    98.379577
   101.478552
   114.969955
   363.597653
 ... Aggregate rates RHS [ns-1]
  1 -0.008838  0.000905  0.000419  0.000493  0.000784
  2  0.002216 -0.007431  0.001915  0.001905  0.000948
  3  0.001077  0.002009 -0.006770  0.003220  0.000137
  4  0.001639  0.002587  0.004170 -0.006181  0.000376
  5  0.003907  0.001930  0.000267  0.000563 -0.002246
 ... Aggregate rates RHS [microseconds-1]
   1    -8.838     0.905     0.419     0.493     0.784
   2     2.216    -7.431     1.915     1.905     0.948
   3     1.077     2.009    -6.770     3.220     0.137
   4     1.639     2.587     4.170    -6.181     0.376
   5     3.907     1.930     0.267     0.563    -2.246
... Times=1/rates [ns] with RCUT=  0.000100
   1      113.14     1104.56     2384.19     2028.74     1274.95
   2      451.29      134.57      522.31      525.02     1054.37
   3      928.48      497.85      147.71      310.54     7275.60
   4      610.18      386.49      239.83      161.78     2659.20
   5      255.98      518.13     3751.02     1775.14      445.18
```

Properties

| # | NHB | RMSDH | Pop | $\Delta G, kcal/mol$ | Ns | List |
|---|---|---|---|---|---|---|
| 1 | 4.7 | 4.5 | 0.0698900 | 0.9565 | 2 | [5-6] |
| 2 | 7.2 | 3.4 | 0.1709892 | 0.4232 | 3 | [7-9] |
| 3 | 12.6 | 0.4 | 0.1792324 | 0.3951 | 2 | [13-14] |
| 4 | 10.2 | 1.7 | 0.2321504 | 0.2409 | 3 | [10-12] |

5 0.5 5.9 0.3477381 0.0000 4 [1-4]

**Ala21. Number of microstates $N_c$ = 20, lagtime $\tau_l = 80\ ns$**

Table S6d.

Comparison of results from full K matrix (dimension $N_c$ = 34) and reduced dimensionality models with dimensions N=2-5. Relaxation times, ns.

| Full K matrix | N=2 | N=3 | N=4 | N=5 |
|---|---|---|---|---|
| 471 | 414 | 365 | 444 | 458 |
| 162 | | 154 | 153 | 157 |
| 104 | | | 100 | 99 |
| 89 | | | | 87 |
| 84 | | | | |

Table S7d. ODR models for ALA21

N=2

... Lifetimes -1/EV(I) [ns], I=1,NS-1
  414.226821
... Aggregate rates RHS [ns-1]
  1 -0.002135 0.000279
  2 0.002135 -0.000279
... Aggregate rates RHS [microseconds-1]
  1 -2.135 0.279
  2 2.135 -0.279
... Times=1/rates [ns] with RCUT= 0.000050
  1 468.34 3585.04
  2 468.34 3585.04

Properties

| # | NHB | RMSDH | Pop | $\Delta G, kcal/mol$ | Ns | List |
|---|---|---|---|---|---|---|
| 1 | 1.6 | 7.6 | 0.1155000 | 1.2136 | 6 | [1-6] |
| 2 | 14.4 | 2.8 | 0.8845000 | 0.0000 | 14 | [7-20] |

N=3

... Lifetimes -1/EV(I) [ns], I=1,NS-1
  154.344004
  365.536049
... Aggregate rates RHS [ns-1]
  1 -0.002630 0.000749 -0.000095
  2 0.003229 -0.004132 0.002313
  3 -0.000598 0.003383 -0.002218
... Aggregate rates RHS [microseconds-1]
  1 -2.630 0.749 -0.095
  2 3.229 -4.132 2.313
  3 -0.598 3.383 -2.218
... Times=1/rates [ns] with RCUT= 0.000050
  1 380.20 1334.28 10528.14

| | | | |
|---|---|---|---|
| 2 | 309.73 | 242.00 | 432.32 |
| 3 | 1671.06 | 295.61 | 450.84 |

Properties

| # | NHB | RMSDH | Pop | $\Delta G, kcal/mol$ | Ns | List |
|---|---|---|---|---|---|---|
| 1 | 0.6 | 7.8 | 0.0861347 | 1.0973 | 4 | [1-4] |
| 2 | 10.1 | 5.2 | 0.3711222 | 0.2266 | 10 | [5-14] |
| 3 | 16.8 | 1.4 | 0.5427431 | 0.0000 | 6 | [15-20] |

N=4

... Lifetimes -1/EV(I) [ns], I=1,NS-1

99.795381
152.675205
443.685978

... Aggregate rates RHS [ns-1]

| | | | | |
|---|---|---|---|---|
| 1 | -0.003207 | 0.002153 | 0.000076 | -0.000014 |
| 2 | 0.002953 | -0.007322 | 0.001411 | 0.000133 |
| 3 | 0.000340 | 0.004592 | -0.005297 | 0.002852 |
| 4 | -0.000086 | 0.000578 | 0.003810 | -0.002970 |

... Aggregate rates RHS [microseconds-1]

| | | | | |
|---|---|---|---|---|
| 1 | -3.207 | 2.153 | 0.076 | -0.014 |
| 2 | 2.953 | -7.322 | 1.411 | 0.133 |
| 3 | 0.340 | 4.592 | -5.297 | 2.852 |
| 4 | -0.086 | 0.578 | 3.810 | -2.970 |

... Times=1/rates [ns] with RCUT= 0.000050

| | | | | |
|---|---|---|---|---|
| 1 | 311.79 | 464.51 | 13116.72 | 0.00 |
| 2 | 338.65 | 136.57 | 708.73 | 7527.38 |
| 3 | 2938.45 | 217.78 | 188.78 | 350.68 |
| 4 | 11643.45 | 1731.23 | 262.47 | 336.69 |

Properties

| # | NHB | RMSDH | Pop | $\Delta G, kcal/mol$ | Ns | List |
|---|---|---|---|---|---|---|
| 1 | 0.4 | 7.9 | 0.0781279 | 1.0646 | 3 | [1-3] |
| 2 | 6.2 | 6.4 | 0.1072131 | 0.8759 | 6 | [4-9] |
| 3 | 12.1 | 4.4 | 0.3486709 | 0.1729 | 6 | [10-15] |
| 4 | 17.3 | 1.0 | 0.4659881 | 0.0000 | 5 | [16-20] |

N=5

... Lifetimes -1/EV(I) [ns], I=1,NS-1

87.005393
98.266552
156.971077
458.221589

... Aggregate rates RHS [ns-1]

| | | | | | |
|---|---|---|---|---|---|
| 1 | -0.003378 | 0.003866 | 0.000725 | 0.000049 | -0.000007 |
| 2 | 0.001851 | -0.009345 | 0.000976 | 0.000176 | 0.000024 |
| 3 | 0.001396 | 0.003923 | -0.007446 | 0.002385 | 0.000481 |
| 4 | 0.000170 | 0.001259 | 0.004254 | -0.007011 | 0.002533 |
| 5 | -0.000039 | 0.000297 | 0.001491 | 0.004401 | -0.003031 |

... Aggregate rates RHS [microseconds-1]

| | | | | | |
|---|---|---|---|---|---|
| 1 | -3.378 | 3.866 | 0.725 | 0.049 | -0.007 |
| 2 | 1.851 | -9.345 | 0.976 | 0.176 | 0.024 |
| 3 | 1.396 | 3.923 | -7.446 | 2.385 | 0.481 |
| 4 | 0.170 | 1.259 | 4.254 | -7.011 | 2.533 |
| 5 | -0.039 | 0.297 | 1.491 | 4.401 | -3.031 |

... Times=1/rates [ns] with RCUT= 0.000050

| | | | | | |
|---|---|---|---|---|---|
| 1 | 296.06 | 258.67 | 1378.81 | 0.00 | 0.00 |
| 2 | 540.16 | 107.01 | 1024.31 | 5692.92 | 0.00 |
| 3 | 716.57 | 254.93 | 134.30 | 419.32 | 2079.38 |
| 4 | 5889.60 | 794.27 | 235.07 | 142.64 | 394.82 |
| 5 | 0.00 | 3363.15 | 670.88 | 227.23 | 329.92 |

Properties

| # | NHB | RMSDH | Pop | $\Delta G, kcal/mol$ | Ns | List |
|---|---|---|---|---|---|---|
| 1 | 0.4 | 7.9 | 0.0781279 | 1.0646 | 3 | [1-3] |
| 2 | 4.3 | 6.9 | 0.0373721 | 1.5042 | 3 | [4-6] |
| 3 | 8.5 | 5.7 | 0.1503703 | 0.6743 | 5 | [7-11] |
| 4 | 12.8 | 4.1 | 0.2681416 | 0.3294 | 4 | [12-15] |
| 5 | 17.3 | 1.0 | 0.4659881 | 0.0000 | 5 | [16-20] |

**Transition Path Theory**

We used Transition Path Theory (TPT) to calculate committors along the folding paths of the ALAn peptides[1, 4]. The committor value $q_k$ at state k is the probability that a trajectory leaving the reactant/initial state and currently at state k will first reach the product/initial state. Here we use the properties that q = 0 for the initial state, q = 1 for the final state and q=0.5 for the transition state of a reaction. The transition matrices and committors are calculated using EMMA tools[4].

Table S8a. ALA5 committor values from full 4x4 transition matrix, reactant is NHB=0, product NHB=3. 2 M – this work, 0 M – results from simulations in water, ref. [6].

| 2 M | | 0 M | |
|---|---|---|---|
| NHB | q | NHB | q |
| [0] | 0.0 | [0] | 0.0 |
| [1] | 0.081 | [1] | 0.054 |
| [2] | 0.218 | [2] | 0.146 |
| [3] | 1.0 | [3] | 1.0 |

Table S8b. ALA8 committor values from N=4 ODR model, reactant is state [0] with NHB=0, product is microstate [6] with NHB=6. 0 M – results from simulations in water, ref. [6].

| 2 M | | 0 M | |
|---|---|---|---|
| NHB | q | NHB | q |
| [0 1] | 0.0 | [0] | 0.0 |
| [2 3] | 0.139 | [1 2 3] | 0.106 |
| [4 5] | 0.344 | [4] | 0.517 |

| | | | |
|---|---|---|---|
| [6] | 1.0 | [5 6] | 1.0 |

ALA8 committor values in full microstate space, N=7

| 2 M | | 0 M | |
|---|---|---|---|
| NHB | q | NHB | q |
| 0 | 0.0 | 0 | 0.0 |
| 1 | 0.062 | 1 | 0.042 |
| 2 | 0.108 | 2 | 0.101 |
| 3 | 0.186 | 3 | 0.227 |
| 4 | 0.301 | 4 | 0.426 |
| 5 | 0.404 | 5 | 0.621 |
| 6 | 1.0 | 6 | 1.0 |

Table S8c. ALA15 committor values from N=5 ODR model, reactant is aggregate state [0 1 2 3] with NHB=0-3, product is aggregate state [12 13] with NHB=12-13. 0 M – results from simulations in water, ref. [6].

| 2 M | | 0 M | |
|---|---|---|---|
| NHB | q | NHB | q |
| [4 5] | 0.405 | [3 4 5] | 0.266 |
| [6 7 8] | 0.576 | [6 7] | 0.532 |
| [12 13] | 1.0 | [11 12 13] | 1.0 |
| [9 10 11] | 0.731 | [8 9 10] | 0.733 |
| [0 1 2 3] | 0.0 | [0 1 2] | 0.0 |

ALA15 committor values in full microstate space, N=14

| 2 M | | 0 M | |
|---|---|---|---|
| NHB | q | NHB | q |
| 0 | 0.0 | 0 | 0.0 |
| 1 | 0.034 | 1 | 0.169 |
| 2 | 0.069 | 2 | 0.205 |
| 3 | 0.135 | 3 | 0.262 |
| 4 | 0.209 | 4 | 0.340 |
| 5 | 0.306 | 5 | 0.407 |
| 6 | 0.399 | 6 | 0.461 |
| 7 | 0.456 | 7 | 0.508 |
| 8 | 0.527 | 8 | 0.556 |
| 9 | 0.588 | 9 | 0.611 |
| 10 | 0.656 | 10 | 0.632 |
| 11 | 0.700 | 11 | 0.647 |
| 12 | 0.742 | 12 | 0.650 |
| 13 | 1.0 | 13 | 1.0 |

Table S8d. ALA21 committor values from N=4 ODR model, reactant is aggregate state [0 1 2] with NHB=0-2, product is aggregate state [15 16 17 18 19] with NHB=15-19. 0 M – results from simulations in water, ref. [6].

| 2 M | | 0 M | |
|---|---|---|---|
| NHB | q | NHB | q |
| [3 4 5 6 7 8] | 0.642 | [3 4 5 6 7 8 9] | 0.504 |
| [9 10 11 12 13 14] | 0.892 | [10 11 12 13 14] | 0.798 |
| [15 16 17 18 19] | 1.0 | [15 16 17 18 19] | 1.0 |
| [0 1 2] | 0.0 | [0 1 2] | 0.0 |

ALA21 committor values in full microstate space, N=20

| 2 M | | 0 M | |
|---|---|---|---|
| NHB | q | NHB | q |
| 0 | 0.0 | 0 | 0.0 |
| 1 | 0.223 | 1 | 0.121 |
| 2 | 0.302 | 2 | 0.149 |
| 3 | 0.407 | 3 | 0.198 |
| 4 | 0.477 | 4 | 0.240 |
| 5 | 0.519 | 5 | 0.289 |
| 6 | 0.618 | 6 | 0.337 |
| 7 | 0.677 | 7 | 0.380 |
| 8 | 0.712 | 8 | 0.419 |
| 9 | 0.755 | 9 | 0.466 |
| 10 | 0.781 | 10 | 0.494 |
| 11 | 0.799 | 11 | 0.521 |
| 12 | 0.819 | 12 | 0.549 |
| 13 | 0.834 | 13 | 0.568 |
| 14 | 0.852 | 14 | 0.590 |
| 15 | 0.871 | 15 | 0.612 |
| 16 | 0.882 | 16 | 0.621 |
| 17 | 0.887 | 17 | 0.629 |
| 18 | 0.889 | 18 | 0.632 |
| 19 | 1.0 | 19 | 1.0 |

**Hydrogen bond patterns**

In general, a peptide with a total of n hydrogen bonds can have a total of $2^n$ states corresponding to formed/broken bonds, among which are $n!/[k!\,(n-k)!]$ arrangements (or patterns) with k hydrogen bonds formed. These patterns are summarized below. For each peptide the maximum number of helical hydrogen bonds n is given. Pattern strings show 0 for broken and 1 for formed bonds, in order from left to right, i.e. '110' denotes a state with formed h-bonds 1 and 2, and broken h-bond 3. The patterns are broken up by number of formed hydrogen bonds, NHB. For each NHB value, the number of patterns found in the MD trajectories and the maximum possible number of patterns, $n!/[k!\,(n-k)!]$, and population fraction are given.

Table S9a. HB pattern population table for ALA5, with n=3 and $2^n = 8$ (2 M proline)
...HB patterns for NHB=0 out of 1 total found and 1 possible
000 0.934696
...HB patterns for NHB=1 out of 3 total found and 3 possible
001 0.018711
100 0.016182
010 0.006217
...HB patterns for NHB=2 out of 3 total found and 3 possible
011 0.005538
110 0.005094
101 0.000923
...HB patterns for NHB=3 out of 1 total found and 1 possible
111 0.012640

Table S9b. HB pattern population table for ALA8, with n=6 and $2^n = 64$ (2 M proline)
...HB patterns for NHB=0 out of 1 total found and 1 possible
000000 0.781954
...HB patterns for NHB=1 out of 6 total found and 6 possible
000001 0.015183
100000 0.011923
001000 0.009457
000100 0.008976
010000 0.007795
000010 0.007631
...HB patterns for NHB=2 out of 15 total found and 15 possible
110000 0.009516
000011 0.008782
001100 0.005233
000110 0.004302
011000 0.003695
101000 0.000973
000101 0.000476
001010 0.000323
100001 0.000321
001001 0.000267
...HB patterns for NHB=3 out of 20 total found and 20 possible
111000 0.011050
000111 0.009902
001110 0.003786
011100 0.002484
110100 0.000615
001101 0.000551
101100 0.000358
001011 0.000356
011010 0.000200
100011 0.000194

...HB patterns for NHB=4 out of 15 total found and 15 possible
001111 0.011815
111100 0.010669
011110 0.002605
111010 0.000666
101110 0.000273
110110 0.000244
111001 0.000239
100111 0.000218
011011 0.000218
011101 0.000218
...HB patterns for NHB=5 out of 6 total found and 6 possible
111110 0.012629
011111 0.008523
111101 0.001056
101111 0.000818
111011 0.000659
110111 0.000466
...HB patterns for NHB=6 out of 1 total found and 1 possible
111111 0.040481

Table S9c.HB pattern population table for ALA15, with n=13 and $2^n = 8192$ (2 M proline) (maximum of top 10 patterns with largest populations shown)
...HB patterns for NHB=0 out of 1 total found and 1 possible
0000000000000 0.252415
...HB patterns for NHB=1 out of 13 total found and 13 possible
0000001000000 0.004938
0000000000001 0.004704
1000000000000 0.004685
0000000001000 0.003824
0001000000000 0.003456
0000100000000 0.003210
0000010000000 0.003139
0000000010000 0.002747
0010000000000 0.002557
0000000000010 0.002550
...HB patterns for NHB=2 out of 78 total found and 78 possible
0000000000011 0.003320
0001100000000 0.002537
1100000000000 0.002222
0000000011000 0.002206
0000000001100 0.002110
0000110000000 0.001808
0000001100000 0.001802
0000011000000 0.001793
0011000000000 0.001239
0000000110000 0.001190
...HB patterns for NHB=3 out of 278 total found and 286 possible

```
0000000000111    0.002690
1110000000000    0.002642
0000111000000    0.002195
0001110000000    0.001835
0000011100000    0.001500
0000000011100    0.001481
0000000111000    0.001262
0000000001110    0.001176
0000001110000    0.001140
0011100000000    0.001006
...HB patterns for NHB=3 out of   278 total found and  286 possible
0000000000111    0.002690
1110000000000    0.002642
0000111000000    0.002195
0001110000000    0.001835
0000011100000    0.001500
0000000011100    0.001481
0000000111000    0.001262
0000000001110    0.001176
0000001110000    0.001140
0011100000000    0.001006
...HB patterns for NHB=4 out of   557 total found and  715 possible
0000000001111    0.003835
0011110000000    0.002758
0001111000000    0.002699
0000000111100    0.002672
0000111100000    0.002496
1111000000000    0.002441
0000001111000    0.002386
0000011110000    0.001630
0000000011110    0.001373
0111100000000    0.001054
...HB patterns for NHB=5 out of   787 total found and  1287 possible
1111100000000    0.005457
0000000011111    0.004598
0011111000000    0.004144
0001111100000    0.003499
0000111110000    0.003240
0000001111100    0.003106
0000011111000    0.002897
0000000111110    0.002516
0111110000000    0.001715
1111010000000    0.000407
...HB patterns for NHB=6 out of   907 total found and  1716 possible
1111110000000    0.009079
0000000111111    0.008649
0000111111000    0.004041
0000001111110    0.004034
```

0001111110000 0.003645
0011111100000 0.003501
0000011111100 0.003136
0111111000000 0.002665
1111101000000 0.000556
0000001111101 0.000335
...HB patterns for NHB=7 out of 867 total found and 1716 possible
0000001111111 0.012893
1111111000000 0.011977
0001111111000 0.006355
0000111111100 0.004585
0111111100000 0.003908
0011111110000 0.003805
0000011111110 0.003407
1111110100000 0.000800
1011111100000 0.000359
0011111101000 0.000302
...HB patterns for NHB=8 out of 781 total found and 1287 possible
1111111100000 0.017344
0000011111111 0.010871
0011111111000 0.006772
0001111111100 0.006611
0111111110000 0.005254
0000111111110 0.003920
1111111010000 0.000957
1000001111111 0.000802
1111100000111 0.000481
1011111110000 0.000418
...HB patterns for NHB=9 out of 561 total found and 715 possible
1111111110000 0.020842
0000111111111 0.012767
0011111111100 0.008572
0001111111110 0.006741
0111111111000 0.006583
1111111101000 0.001149
0001111111101 0.000571
1011111111000 0.000522
0011111111010 0.000460
1111000011111 0.000376
...HB patterns for NHB=10 out of 276 total found and 286 possible
1111111111000 0.026285
0001111111111 0.021577
0011111111110 0.009253
0111111111100 0.007423
1111111110100 0.001528
0011111111101 0.000811
1011111111100 0.000675
0010111111111 0.000495

1111111101100    0.000480
0011111111011    0.000467
...HB patterns for NHB=11 out of   78 total found and  78 possible
1111111111100    0.034161
0011111111111    0.030444
0111111111110    0.008949
1111111111010    0.001789
0111111111101    0.000766
1111111111001    0.000736
1011111111110    0.000714
1111111110110    0.000498
1001111111111    0.000498
0111111111011    0.000444
...HB patterns for NHB=12 out of   13 total found and  13 possible
1111111111110    0.035874
0111111111111    0.028596
1111111111101    0.003089
1011111111111    0.002312
1111111111011    0.001822
1101111111111    0.001099
1111111110111    0.000949
1110111111111    0.000810
1111111101111    0.000786
1111111011111    0.000733
...HB patterns for NHB=13 out of   1 total found and  1 possible
1111111111111    0.116499

Table S9d. HB pattern population table for ALA21, with n=19 and $2^n = 524{,}288$ (2 M proline)(maximum of top 10 patterns with largest populations shown; selected patterns close to coil, midpoint and helix states)
...HB patterns for NHB=0 out of   1 total found and  1 possible
0000000000000000000    0.056317
...HB patterns for NHB=1 out of   19 total found and  19 possible
0000000000000000001    0.001232
1000000000000000000    0.001037
0000001000000000000    0.001030
0000010000000000000    0.000953
0000000000000100000    0.000880
0000000000000001000    0.000842
0000000000010000000    0.000722
0000100000000000000    0.000704
0000000000000000100    0.000655
0001000000000000000    0.000654
...HB patterns for NHB=2 out of   171 total found and  171 possible
1100000000000000000    0.000617
0000000000000000011    0.000608
0011000000000000000    0.000506
0000000000000000110    0.000419
0000000000011000000    0.000359

0000000000000001100    0.000335
0000110000000000000    0.000326
0000001100000000000    0.000322
0000011000000000000    0.000308
0000000001100000000    0.000299
...HB patterns for NHB=3 out of   738 total found and  969 possible
0000000000000000111    0.000946
0011100000000000000    0.000483
0111000000000000000    0.000457
0000001110000000000    0.000428
1110000000000000000    0.000418
0000000111000000000    0.000295
0000000001110000000    0.000245
0000000000000001110    0.000232
0000111000000000000    0.000230
0001110000000000000    0.000216
…
...HB patterns for NHB=7 out of   4194 total found and  50388 possible
1111111000000000000    0.002839
0000011111110000000    0.002720
0000000011111110000    0.001548
0000000000001111111    0.001518
0000000001111111000    0.001384
0000000111111100000    0.001350
0000111111100000000    0.001138
0001111111000000000    0.000989
0111111100000000000    0.000979
0000001111111000000    0.000965
...HB patterns for NHB=8 out of   5396 total found and  75582 possible
1111111100000000000    0.003327
0000001111111100000    0.002022
0000011111111000000    0.001975
0000000000011111111    0.001925
0001111111100000000    0.001707
0000000001111111100    0.001637
0000000111111110000    0.001431
0000000011111111000    0.001119
0011111111000000000    0.000924
0000111111110000000    0.000913
...HB patterns for NHB=9 out of   6224 total found and  92378 possible
1111111110000000000    0.003211
0000000000111111111    0.002656
0000000011111111100    0.002265
0011111111100000000    0.002161
0000001111111110000    0.002024
0000000111111111000    0.002022
0000011111111100000    0.001990
0111111111000000000    0.001662

0000000001111111110    0.001613
0000111111111000000    0.001339
...HB patterns for NHB=10 out of   6623 total found and  92378 possible
0000000001111111111    0.005376
1111111111000000000    0.004821
0000001111111111000    0.003543
0000011111111110000    0.002781
0000000011111111110    0.002770
0111111111100000000    0.002363
0011111111110000000    0.001966
0001111111111000000    0.001924
0000000111111111100    0.001860
0000111111111100000    0.001772
...HB patterns for NHB=11 out of   6368 total found and  75582 possible
1111111111100000000    0.010454
0000000011111111111    0.008962
0000111111111110000    0.004065
0000001111111111100    0.003593
0000011111111111000    0.003483
0001111111111100000    0.002547
0000000111111111110    0.002373
0011111111111000000    0.002054
0111111111110000000    0.001815
0001111100000111111    0.000671
…
...HB patterns for NHB=16 out of   927 total found and  969 possible
0001111111111111111    0.024359
1111111111111111000    0.024118
0011111111111111110    0.011192
0111111111111111100    0.008616
1111111111111110100    0.001567
0011111111111111101    0.000979
1011111111111111100    0.000712
0010111111111111111    0.000627
0011111111111111011    0.000573
0111111111111111010    0.000514
...HB patterns for NHB=17 out of   171 total found and  171 possible
0011111111111111111    0.036476
1111111111111111100    0.035018
0111111111111111110    0.010079
1111111111111111010    0.002053
0111111111111111101    0.000878
1011111111111111110    0.000817
1111111111111111001    0.000783
1111111111111110110    0.000583
1001111111111111111    0.000541
0111111111111111011    0.000516
...HB patterns for NHB=18 out of   19 total found and  19 possible

1111111111111111110    0.040320
0111111111111111111    0.032871
1111111111111111101    0.003513
1011111111111111111    0.002638
1111111111111111011    0.002076
1101111111111111111    0.001274
1111111111111110111    0.001088
1110111111111111111    0.000933
1111111111111101111    0.000891
1111111111110111111    0.000848
...HB patterns for NHB=19 out of   1 total found and  1 possible
1111111111111111111    0.131058

## Diffusion and friction

Diffusion coefficients are calculated using the method of Bicout and Szabo [7], which converts a kinetic equation into a Smoluchowski equation for diffusion on a one-dimensional free energy landscape. For a helix propagation step from $i$ to $i+1$ hydrogen bonds, the diffusion coefficient is $D_{i+1/2} = d^2 k_{i+1,i}(p_i/p_{i+1})^{1/2}$ with $k_{i+1,i}$ the rate constant for hydrogen bond count increase from $i$ to $i+1$, $p_i$ is the probability of finding $i$ hydrogen bonds and $d$ is the average peptide length change for this process. Corresponding friction coefficients f are calculated through the Einstein relation, $f = k_B T/D$, with $k_B$ the Boltzmann constant and T the absolute temperature. The results are presented in Fig. S1 below.

The calculated values of D and f crucially depend on the parameter $d$, the change in peptide length upon addition or removal of a single hydrogen bond. The most straightforward approach is to use the standard helix rise per residue, which corresponds to $d$=0.15 nm. We use this value here, as it has been employed previously [8].

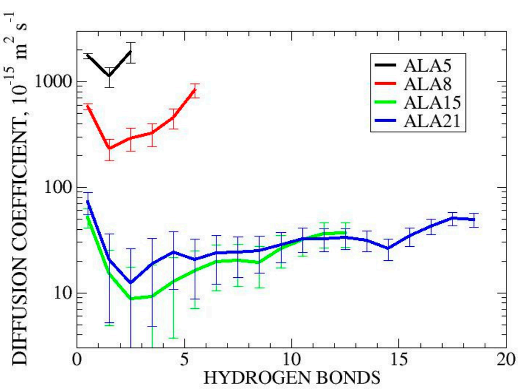

A. Diffusion coefficients

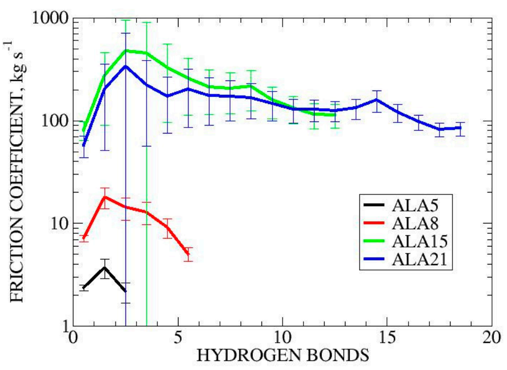

B. Friction coefficients

Figure S1. Diffusion and friction for helix propagation, calculated from kinetic rate constants. Shown are D and f values for NHB transitions from $i$ to $i + 1$, averaged in forward and reverse direction.

## System Composition

**Table S10.** System compositions. Pep – peptide atoms, Tot – total atoms, Wat – water molecules, Na+ - sodium ions, Cl- - chloride ions, Pro – proline zwitterions, a – cubic box size, nm and c is the concentration in M= mol/L.

| System | Pep | Tot | Wat | Na+ | Cl- | Pro | a | c |
|---|---|---|---|---|---|---|---|---|
| ALA5 a[1] | 59 | 3982 | 1050 | 4 | 4 | 38 | 3.18 | 1.97 |
| ALA5 e[1] | 59 | 3381 | 890 | 3 | 3 | 45 | 3.37 | 1.95 |
| ALA8 a[1] | 89 | 4162 | 1167 | 4 | 4 | 50 | 3.49 | 1.96 |
| ALA8 e[1] | 89 | 4448 | 1167 | 4 | 4 | 50 | 3.48 | 1.97 |
| ALA15 a[2] | 159 | 8527 | 2240 | 8 | 8 | 96 | 4.33 | 1.96 |
| ALA15 b[2] | 159 | 8527 | 2240 | 8 | 8 | 96 | 4.32 | 1.98 |
| ALA15 c[2] | 159 | 8527 | 2240 | 8 | 8 | 96 | 4.33 | 1.97 |
| ALA15 d[2] | 159 | 8527 | 2240 | 8 | 8 | 96 | 4.33 | 1.97 |
| ALA15 e[2] | 159 | 8527 | 2240 | 8 | 8 | 96 | 4.33 | 1.96 |
| ALA21 a[2] | 219 | 12599 | 3314 | 12 | 12 | 142 | 4.94 | 1.96 |
| ALA21 b[2] | 219 | 12681 | 3335 | 13 | 13 | 143 | 4.94 | 1.97 |
| ALA21 c[2] | 219 | 13031 | 3429 | 13 | 13 | 147 | 4.99 | 1.97 |
| ALA21 d[2] | 219 | 12770 | 3359 | 13 | 13 | 144 | 4.95 | 1.98 |
| ALA21 e[2] | 219 | 12770 | 3359 | 16 | 16 | 144 | 4.95 | 1.98 |

[1] a – trajectory started from helix, e – trajectory started from extended conformation.
[2] a-e are five independent MD trajectories generated as described below.
Extended conformation trajectories were started in larger solvent box and after a 10 ns NPT equilibration, peptide was re-solvated in a smaller solvent box, comparable in size to the one obtained for start from helix. For ALA15 and ALA21, separate 1,000 ns MD trajectories starting from helix were generated at 500 K. After RMSD clustering, three different cluster centers were selected to start the 300 K MD simulations b, c, d; a and e had same starting structures as before – helix and extended.

## References


(1) Bowman, G. R.; Noé, F.; Pande, V. S. *An Introduction to Markov State Models and Their Application to Long Timescale Molecular Simulation*, 1st ed.; Springer Netherlands : Imprint: Springer,, 2014. DOI: 10.1007/978-94-007-7606-7.
(2) Buchete, N. V.; Hummer, G. Coarse master equations for peptide folding dynamics. *J Phys Chem B* **2008**, *112* (19), 6057-6069. DOI: Doi 10.1021/Jp0761665.

(3) Kube, S.; Weber, M. A coarse graining method for the identification of transition rates between molecular conformations. *J Chem Phys* **2007**, *126* (2). DOI: Artn 024103 10.1063/1.2404953.
(4) Senne, M.; Trendelkamp-Schroer, B.; Mey, A. S. J. S.; Schutte, C.; Noe, F. EMMA: A Software Package for Markov Model Building and Analysis. *J Chem Theory Comput* **2012**, *8* (7), 2223-2238. DOI: Doi 10.1021/Ct300274u.
(5) Hummer, G.; Szabo, A. Optimal Dimensionality Reduction of Multistate Kinetic and Markov-State Models. *J Phys Chem B* **2015**, *119* (29), 9029-9037, Research Support, N.I.H., Intramural Research Support, Non-U.S. Gov't. DOI: 10.1021/jp508375q.
(6) K. Kuczera, R. S. a. G. S. J. Microscopic effects of proline co-solvent on alanine homopeptide structure, solvation and helix folding dynamics. *Journal of Biomolecular Structure and Dynamics* **2025**, *submitted*.
(7) Bicout, D. J.; Szabo, A. Electron transfer reaction dynamics in non-Debye solvents. *J Chem Phys* **1998**, *109* (6), 2325-2338. DOI: Doi 10.1063/1.476800.
(8) Swiatek, A.; Kuczera, K.; Szoszkiewicz, R. Effects of Proline on Internal Friction in Simulated Folding Dynamics of Several Alanine-Based α-Helical Peptides. *J Phys Chem B* **2024**. DOI: 10.1021/acs.jpcb.4c00623.
